# Protecting Women’s and Newborns’ Rights in a Public Maternity Unit During the COVID-19 Outbreak: The Case of *Dra. Eloísa Díaz - La Florida* Hospital in Santiago, Chile

**DOI:** 10.3389/fsoc.2021.614021

**Published:** 2021-03-02

**Authors:** Gonzalo Leiva, Michelle Sadler, Camila López, Susana Quezada, Víctor Flores, Cristian Sierra, Susan Díaz, Christian Figueroa

**Affiliations:** ^1^ Maternity Dra. Eloísa Díaz - La Florida Hospital, Santiago, Chile; ^2^ Faculty of Medicine, Pontificia Universidad Católica de Chile, Santiago, Chile; ^3^ Chilean Observatory of Obstetric Violence, Santiago, Chile; ^4^ Faculty of Liberal Arts, Universidad Adolfo Ibáñez, Santiago, Chile; ^5^ International Consultant, Early Childhood Development and Maternal and Child Health, Washington, DC, United States

**Keywords:** humanistic childbirth, human rights in childbirth, COVID-19, public health, midwifery

## Abstract

The Maternity in Dra. Eloísa Díaz’ hospital, located in the municipality of La Florida and city of Santiago, Chile, opened its doors in 2014, and has integrated a humanistic model of care called the “Safe Model of Personalized Childbirth” since 2016. With around 3,000 births per year, it has been recognized as an example of excellence in maternity care in the country. The COVID-19 outbreak presented a big challenge to this Maternity: to maintain its quality of care standards despite the health crisis. This article presents the Maternity’s responses to the pandemic from March to July 2020, describing the strategies that were deployed and the obstetric outcomes achieved. Semi-structured interviews with midwives and OB-GYNs, and a retrospective review of the childbirth standards of care and outcomes of the 55 women who tested positive for SARS-CoV-2, were carried out. The results show how the Maternity's staff responded in order to avoid a significant negative impact on the rights of women and newborns. Protocols to reestablish the companion during labor and childbirth and skin-to-skin contact, which were suspended for almost three weeks at the beginning of the outbreak, and the creation of an Instagram account to communicate with the external community were some of the measures taken. After some initial weeks of adjustment, the standards of care for all women, included for those diagnosed with COVID-19, were reestablished almost to pre-pandemic levels. This case shows that quality of care can be maintained and the rights of women and newborns can be respected during health crisis such as the COVID-19 pandemic.

## Introduction

### Chile and Its Healthcare System

Chile is a democratic republic located in South America, with a population of 17,574,003 inhabitants in 2017 (National Institute of Statistics, 2018). It is considered a high-income country and had a Gross Domestic Product (GDP) per capita of US$25,041 in 2019 (World Bank, 2020). During 2017, 8.6% of its population lived in poverty and 0.3% lived on less than $1.90USD per day (Ministry of Social Development and Family, 2018; World Bank, 2020). However, more than 30% of the population is economically vulnerable and income inequality remains high (World Bank, 2020). The Chilean health system has maintained the structure that was defined during the 1980s (ECLAC, 2012), which consists of both public and private sector insurance and care provision, funded through social contributions (payroll taxes), general taxes, co-payments and voluntary premiums. The total per capita health expenditure was US$2,159 in 2019, representing 9.1% of the country’s GDP. Public spending represented 5.4% of GDP and out-of-pocket spending accounted for 33.9% of health expenditures (OECD, 2020). Public provision of health services is highly decentralized and is coordinated under a National Health Services System, which consists of 29 Health Services (*Servicios de Salud*), geographically distributed. Health insurance is provided by private insurers (ISAPREs) and mostly by the National Public Health Insurance Fund (FONASA), which insured 78% of the population in 2017. The public sector, unlike the private, is characterized by hospital infrastructure deficits, low privacy in patient care, impersonality in the treatment of patients, unfavorable working conditions and low wage levels (Goic, 2015). Population wise, 92% of those belonging to the lowest income decile of the population and 80.6% of women are beneficiaries of public insurance (Ministry of Social Development and Family, 2018). FONASA’s beneficiaries have access to two different health care plans determined by household income and number of family members: Institutional Care Modality (MAI) and Free Choice Modality (MLE). Regarding birth, the MAI does not require any extra out-of-pocket expenditure, and the MLE does entail out-of-pocket expenditure, which in September 2020 was around US$360 (National Public Health Insurance Fund, 2020).

In 2017, 219,186 live births were registered in the country; skilled birth attendance coverage was 99.7% (National Institute of Statistics, 2019); the IMR (infant mortality ratio; infant deaths/1,000 live births) was 6/1,000 (UNICEF, 2018); and the MMR (maternal mortality ratio, maternal deaths/100,000 live births) was 13/100,000 (WHO, 2019). Although these are good maternal health indicators, they hide large gaps in access and quality of health care between private and public health facilities, disparities between territories and lack of options regarding place of birth. Only facility births are covered by insurance, and home birth, although not illegal, is discouraged and not recognized by the health system.

More than ¾ of the country’s births, 77.4%, occurred in the public health sector in 2017 (Ministry of Health, 2020a). Although the quality of interpersonal care between health personnel and women is reported to be better in the private than in the public sector (OVO Chile, 2018), both health sectors exhibit alarmingly high routine obstetric interventions during childbirth, as indicated by a national cesarean birth rate of 50% in 2015 (National Institute of Human Rights, 2016). A study conducted in nine major public regional maternity hospitals with primiparous and multiparous women who were admitted in labor reported the following interventions: 91% had medically induced/augmented labors, 55% had continuous fetal intrapartum monitoring, 56% had episiotomies, and 80% delivered in the lithotomy position (Binfa et al., 2016). The rate of obstetric interventions is similarly high in private health (OVO Chile, 2018), with cesareans being 27% higher in the private than in the public sector (National Institute of Human Rights, 2016).

Chile is internationally recognized for a scaled-up flagship program for early childhood development called “Chile Grows with You” (Richter et al., 2016), which has had national coverage since 2008. An axis component of this intersectoral policy is the Biopsychosocial Development Support Program (PADB), which, among other objectives, aims to strengthen prenatal care and provide personalized care during childbirth (Ministry of Social Development and Family and World Bank, 2018). Hand-in-hand with this program, in 2008, the Ministry of Health launched the *Manual for Personalized Attention in the Reproductive Process* (Ministry of Health, 2008), with the objective of implementing a humanistic model of birth (Davis-Floyd, 2001; Davis-Floyd, 2018). Although these policies and recommendations have helped to improve some practices, mainly those that constitute indicators of PADB (such as the presence of a companion during labor and birth, and skin-to-skin contact with the newborn for 30 or more minutes immediately after birth), there has not been a profound nation-wide paradigm shift toward the humanization of care during labor and birth. Thus, the health sectoral efforts to improve maternity care during the last decade have had limited impact (Binfa et al., 2016; OVO Chile, 2018).

### SARS-Cov-2 in Chile and Its Threat to Respectful Maternity Care and Birth Rights

In Chile, the first COVID-19 case was reported on March 2, 2020. Exactly two weeks later, on March 16, the Ministry of Health indicated that Chile was entering Phase 4, which corresponds to the stage of sustained community spread of the virus. That same day, schools suspended their activities throughout the national territory, and two municipalities in the Metropolitan Region declared a state of emergency: Santiago and La Florida (Municipality of La Florida, 2020). This decision was made two days before the official publication of Supreme Decree No. 104, which declared a state of constitutional exception of catastrophe, due to public calamity, in the territory of Chile (Ministry of the Interior and Public Security, 2020). On March 22, a curfew began throughout the nation for the population to remain indoors from 22:00 to 05:00. And, since March 26, “dynamic” quarantines have been implemented in territories throughout the country, defining harsher or softer measures in various regions according to their COVID-19 related indicators. This has meant opening some communities while others remain closed, or vice versa, even when these are neighboring territories.

La Florida municipality, which is the fourth largest in the region, with a population of 402,433 and accounting for 5% of the region’s inhabitants (Ministry of Health, 2020b), entered quarantine on May 15 and remained in that state until August 30, 2020, when we first submitted this article.

The Epidemiological Report N° 39, published by the Department of Epidemiology (Ministry of Health, 2020b), reported that on August 2 (5 months after the first reported case), 401,142 people had been infected and 9,968 had died from COVID-19. This placed Chile in the 8^th^ position of diagnosed cases in the world on that date (WHO, 2020a); and as the 3^rd^ country with the highest death rate per million inhabitants, with 53.17 deaths per million, after only the United Kingdom (70.08) and Peru (64.55) (John Hopkins University, 2020).

There was no doubt that COVID-19 would pose a challenge to healthcare systems around the world, but what its specific impact on maternity care would be was unclear. The “Guidelines for pregnant women with suspected SARS-CoV-2 infection” (Favre et al., 2020), published on March 3 suggested shortening labor to avoid exhaustion in woman with confirmed infections. Although stating that there was no evidence of vertical transmission of the virus, the authors recommended the isolation of the newborn for at least 14 days or until viral shedding cleared--time during which breastfeeding was not recommended (Favre et al., 2020). The guidelines, which could have “consequences of unpredictable magnitude in the long term” according to the authors, were translated into Spanish (Martinez-Portilla et al., 2020) and widely disseminated, despite evidence showing the harm those measures could cause (Schmid et al., 2020; Smith, 2020; Stuebe, 2020).

Up to May, according to a review of 15 articles published by 10 scientific societies, including WHO, there was no definitive evidence to suggest vertical transmission of SARS-CoV-2. Likewise, the review concluded that it is not advisable to separate mothers from newborns or to discourage breastfeeding, unless the mother is seriously ill (Narang et al., 2020). But despite the evidence, and the fact that WHO (2020b) had pointed out since early March that pregnant women should have access to specialized, respectful and woman-centered care, as the pandemic spread, voices of alert around the world expressed concern for the rights of women and newborns during childbirth (International Confederation of Midwives, 2020; Human Rights and Childbirth, 2020; Sadler et al., 2020). NGOs denounced that in many settings, women’s labors were being accelerated unnecessarily (programmed induction of labor, routine Pitocin, instrumental deliveries); were given a planned cesarean as their only option for birth; were denied a partner during labor and birth; were routinely separated from their newborns; and were not allowed to breastfeed (Childbirth is Ours, 2020; OVO Chile, 2020).

In our country, the Chilean Pediatric Society published the first guidelines for mothers with COVID-19 and their newborns in mid-March. Skin-to-skin contact was not recommended in women with symptoms, regardless of the severity of the illness; early clamping of the cord was promoted; and breastfeeding was not recommended for COVID-19^+^ mothers. In the third version of this document, published on April 2, some of these measures were revised, recommending skin-to-skin contact and breastfeeding in confirmed or suspected COVID-19 mothers, provided that their babies were full term, and that the mothers were not in serious condition (Chilean Pediatric Society, 2020). It took the Ministry of Health almost 4 months since the first reported COVID case in the country to issue guidelines on maternity care, on June 25a date until which each Health Service and maternity unit was responding to the extent of its local resources and decisions. The Chilean Obstetric Violence Observatory reported that by the end of May, 46% of public hospitals had completely suspended accompaniment during labor and birth, a situation that did not occur in any private clinic in the country (OVO Chile, 2020).

Until June 12, there were only rumors of the existence of technical documents that hadn’t been officially authorized. On that date, the Ordinance N° 1891 on recommendations for postpartum women, boys and girls regarding risk factors of child morbidity and mortality and their mental health in the COVID-19 pandemic was issued, recommending the presence of a companion during birth with the adequate personal protective equipment (PPE), skin-to-skin contact after birth and breastfeeding (Ministry of Health, 2020c). It was only on June 25, after strong pressure from civil society organizations and from the National College of Midwives (Chilean College of Midwives, 2020), that the new “Guidelines for the management of SARS-CoV-2 (COVID-19) cases in pregnant and postpartum women and/or dyads” were published. The document recommends companionship in the cases of women with COVID-19, skin-to-skin contact, and breastfeeding, as the Chilean Pediatric Society had done earlier. Regarding joint sleeping, it recommends making decisions based on each institutional reality (Ministry of Heath, 2020d).

Since the onset of the pandemic, most public maternities throughout the country receded in standards of care that had gradually been improving during the last decade. But a few did not, and made huge efforts to maintain the quality of care that they had achieved. This has been the case of the Maternity of Dra. Eloísa Díaz - La Florida Hospital in Santiago, which has been operating since late 2014 and has been recognized as an example of excellence in maternal care in the country (South-East Metropolitan Healthcare Service, 2020). As a brief context, La Florida municipality had reported 13,357 COVID-19^+^ cases by August 2 (the fourth municipality with most cases in the Metropolitan Region) and 451 deaths (second in the Region) (Ministry of Health, 2020b).

There was a great challenge ahead, the greatest in the short history of the Maternity of Dra. Eloísa Díaz – La Florida Hospital: to face the greatest threat to the populations’ health of which we had a record, while maintaining the standards of care that we had achieved with hard work. And this, in one of the biggest municipalities of the country and one of the hardest hit by the pandemic.

## Methods

We approached the process of describing and systematizing the responses of Dra. Eloísa Díaz’ Maternity to the COVID-19 outbreak between March and July 2020 using mixed methodologies. Qualitative semi-structured interviews were carried out to inquire about the health personnel’s experiences and perceptions with eight key maternity officials: six midwives and two obstetricians. Of the midwives, five were the heads of shifts during the period studied, and one was the main supervisor of all those shifts. One obstetrician was the head of residents, and the second the head OB-GYN of the maternity. All participants signed a consent document, interviews were carried out by one of the authors through Zoom and were recorded, with an average duration of 35 min. The interview guide covered the following topics: impacts of SARS-Cov-2 on the maternity personnel and at a personal level; impacts on the standards of maternity care; opinions of the management’s work and of information and communication during the outbreak; milestones or key elements identified to maintain the previous standards of care; and recommendations for future work. Additionally, four of the midwives in charge of the Maternity’s newly created Instagram account responded to a short interview guide through WhatsApp calls on the following topics: the process that led to the creation of the account, the motivation to participate in the project, and the impact of the account on their work and on the Maternity’s standards of care. Of these four midwives, one had also responded to the semi-structured interview, as she was head of shift and also one of the Instagram volunteers.

All interviews were transcribed verbatim, coded, and analyzed using thematic analysis--a qualitative method that enables thematic patterns to be identified from the collected data (Creswell, 2014). The first three authors transcribed the interviews, read and re-read the transcriptions to ensure accuracy with the recording, and coded the interviews. A priori codes describing broad analytical dimensions were derived from the interview guides, and inductive codes were developed as the data was examined. After the coding process, the other authors joined the following interpretation process, which occurred iteratively, reviewing interpretations and reaching final conclusions. When quoting our interlocutors throughout this article, we will follow a chronological numbering system, with (1)–(8) referring to the semi-structured interviews conducted in that order; and to (I.1)–(I.4) when alluding to the four short interviews that describe the Instagram account.

Despite our initial intention of interviewing women and families who had received care within the Maternity, we instead privileged interviews with health personnel for two main reasons: firstly, in order to gain an in-depth understanding of the decision-making process that occurred in response to the pandemic; and secondly, due to the limited availability of time to conduct research, given that most authors were themselves working as health personnel in the Maternity during the COVID outbreak. In order to include some women’s experiences, the article contains a few testimonies from users posted publicly on the Maternity Instagram account. As well, and regarding the impact of the Instagram account, we carried out a quantitative analysis of the interactions on that account during its first 88 days of operation (May 4–July 31), detailing number of users and interactions, and quoting some of the posts herein.

In order to report the main obstetric outcomes of births within the Maternity, we retrieved data on all births that occurred in the Maternity during 2019 and 2020 (until July 31) from the Hospital’s databases, and conducted a retrospective review of 55 pregnant women with laboratory-confirmed COVID-19 (with maternal throat swab samples that were positive for SARS-CoV-2) who were admitted to the Maternity and gave birth between May 1, 2020, and July 31, 2020. The data on COVID-19^+^ cases was collected by Maternity midwives and systematized in a table of obstetric variables based on the intrapartum set dataset extracted from the database of the American College of Nurse-Midwives (ACNM), which was modified and adapted by the researchers to the local reality and the context of the pandemic. The study protocol was approved by the Dra. Eloísa Díaz Hospital’s Scientific Ethics Committee.

## Results

### Safe Model of Personalized Childbirth in Dra. Eloísa Díaz Hospital

Hospital Dra. Eloísa Díaz is part of the South-East Metropolitan Healthcare Service and is one of the 198 hospitals of varying complexity that make up the public network in the country. The hospital provides care to women enrolled in the nine primary care centers of La Florida. Between 2016 and 2019, an average of 4,470 live births per year were registered in La Florida (Ministry of Health, 2020e), of which an average of 66% took place in the Dra. Eloísa Díaz Maternity. Between January and July 2020, 70% of all births in the municipality occurred in this Maternity (estimated by the authors from municipal data), which attends women insured under the Institutional Care Modality (MAI), which does not require extra out-of-pocket expenditures (besides the insurance program of FONASA).

The gynecobstetric unit on which this article focuses is one of the three units of the Women’s Responsibility Center (henceforth referred to as “Women’s CR”). The unit has four areas: a recovery room with six beds; five surgical wards where vaginal births and cesarean sections, as well as elective and emergency obstetric and gynecological surgeries, take place; a prepartum room with eight beds, where only labor takes place (in the second stage, the laboring women are transferred to a surgical ward); and four Comprehensive Childbirth Rooms inside the surgical ward, where labor and vaginal delivery take place. Women are admitted to the traditional prepartum room and gynecobstetric wards or to the Comprehensive Childbirth Rooms depending on their risk factors and room availability. The unit is organized into two teams, which are referred to as the “medical” and “non-medical” teams. The medical team is composed of 18 ob-gyns organized in groups of three that rotate every six days, being available for the three units of the Women’s RC (emergency, gynecobstetric, and hospitalization). The non-medical team is composed of 103 health staff. Of the total staff, 14 work during the day, from Monday to Friday, and the rest of the team is organized into four shifts made up of: 10 nursing technicians, four service assistants, and eight midwives (called *matronas*, who in Chile follow a direct-entry (non-nursing) program of 5 years of university education). All teams rotate on day and night shifts, and then have two days off (after which the cycle repeats). The security, cleaning, food, and maintenance personnel of the unit are part of a Concessionaire Society.

Despite having opened during late 2014, the Maternity unit began working fully in 2016, when the Comprehensive Childbirth Rooms became functional and the “Safe Model of Personalized Childbirth” was implemented. This model attempts to improve women’s satisfaction, reduce excessive and unnecessary interventions (including cesareans), and improve maternal, fetal, and neonatal outcomes. Since 2018, the unit’s management has included a detailed, monthly analysis of births, which are analyzed according to the place of development of labor. This is done in order to promote the same standards of care of the Comprehensive Childbirth Rooms in the traditional rooms, such as the presence of a companion during labor, free movement and walking, use of non-pharmacological pain relief methods, skin-to-skin contact of 30 min or more, breastfeeding on newborn demand, and reduction of interventions such as use of Pitocin (synthetic oxytocin) and episiotomy. Between May 2016 and July 2020, 3,681 births took place in the Comprehensive Childbirth Rooms. During 2020 (until the end of July), 30% of all hospital births occurred in these rooms.

### Maternity Care During the Pandemic

Anticipating the arrival of the COVID-19 pandemic in Chile, at the beginning of February, all staff at Eloísa Díaz Hospital began to be trained in 27-h courses on standard precautions for infection prevention and use of PPE such as disposable aprons, gloves, masks and face shields. The courses were focused on protecting the health personnel and their clients from infection, and not on technical issues related to pregnancy and delivery.

On March 10, the last available dates for this training were published, when the country was a week away from entering Phase 4 (of sustained spread of the virus), and La Florida of being declared in a state of emergency. By that date, there was little information on the impact of COVID-19 on pregnant and laboring women, and some of the first guidelines that had been published worldwide were recommending retreat from some standards of care that had become rights in the Maternity (companion of choice during labor, skin-to-skin contact after birth, early breastfeeding) (Favre et al., 2020).

The health staff of the maternity reported that the main feeling of those first weeks was of fear: of the unknown, of getting infected, of infecting their family members. The heads of shifts express: “The first thing that comes to my mind is fear; at first no one knew much about what we were going to face, there was a lot of fear of getting infected” (1); “It generated fear at first, because we didn't know what we were dealing with: fear of infecting ourselves, of infecting our families, our users, that a newborn could be infected” (4); “It felt like we were all very scared and that feeling was pretty strong” (5); “There was a lot of fear of the unknown, of how we were going to face this process” (6).

On Monday, March 16, the day the municipalities of Santiago and La Florida entered a state of emergency, the first email was sent by the heads of the unit to the entire team, acknowledging that the scenario was unprecedented nationally and worldwide, and listing the first measures to be implemented: suspending the regular fourth shift and moving to a 24 × 3 scheme (a 24-h shift followed by 3 days off); avoiding kissing and hand to hand greetings; prohibition of arriving to the hospital in uniform (compulsory dressing in uniform in the hospital); and suspension of vacations and training courses. One of the midwives interviewed (head of shift) points out that:

Regarding management, I feel that my bosses understood how to be a boss, how to be a leader, with a focus on the people who worked with them, they knew how to … do everything possible to channel that fear. I feel they acted a bit like midwives: they took the staff, educated them, took all the precautions, so that we felt safe working. (1)

On March 17 at a meeting between the Women’s CR and the staff of the Neonatology Unit, the “Measures to prevent infection by COVID-19 in pregnant women and their newborns” began to be outlined, based on the first recommendations of the Chilean Pediatric Society. At the meeting, it was clearly stated that those first measures, which were very conservative (not recommending skin-to-skin contact or breastfeeding for COVID-19^+^ women), would be modified as more evidence became available. Two days later, the South-East Metropolitan Healthcare Service (SSMSO), which manages and coordinates three large hospitals and the primary health care corresponding to the same territory, issued recommendations for the organization of the system and the use of PPE. The WhatsApp group “COVID 19 SSMSO” was created. The midwifery care coordinator highlights the creation of this network as one of the milestones of the management process:

Communication, not only between internal teams but also between the internal team and the extra-system: primary health care, Health Service and central level. This was essential to be able to agree on ideas and at the same time help each other. (6)

Only three days later, on March 22, we had the first COVID-19 suspicious pregnant woman at the Maternity. This case set off alarms regarding the possibility of preventive quarantines of large numbers of health staff, and made us organize the personnel in a way that would not impact the standards of care, and that at the same time would not entail too great a work overload on the teams. On that day, and coinciding with the start of a national curfew, it was decided that all visits to hospitalized users of the hospital were suspended, including the companion during labor and childbirth.

On April 3, two members of our staff announced the presence of COVID-19 symptoms and took the test in the hospital’s emergency unit; it was the first time that we were forced to review the flow of personnel. Fortunately, both tests came out negative. The experience led to the creation of an executive WhatsApp group composed of the four shift leaders, the two supervisors, and the maternity coordinator (all of them midwives) as an alternative to an existing and more extensive group that included other levels of supervision.

On the following day, we decided that the use of masks was compulsory during the entire shifts and during interactions among staff and with users. Very much disliking the suspension of companionship during childbirth, we started advancing in actions to reverse this measure. From there onwards, we asked for provision of surgical masks for the entire staff, plus all women and their companions. Regarding this participatory means of management, a head of shift expresses:

The way in which we function as a team is outstanding; I feel that every decision that has been made has always included the opinion of the shift heads and that seems good to me in terms of productivity and also because it is a matter of respect and appreciation with our work (…) Each measure that is adopted is made by a supervisor but seen by a clinical team; it makes everything easier because the collaborations are there. (3)

On the newly created WhatsApp group, we immediately began to discuss the best way to organize the staff. On April 13, three proposals were presented and the heads of the unit decided on the 24 × 4 rotary, which entailed dividing the existing four shifts and creating a fifth one. To reduce infection risk, there would be fewer personnel on each shift than before (two nursing technicians, one service assistant and one midwife less than in the previous shifts). One new midwife was hired to match these numbers, and the new organizational model was made official four days later, to begin operating on Monday April 20. The email that communicated these measures explained that “we have been working on these proposals together with the four heads of shift for more than 10 days, to be able to face in the best way possible the scenarios that we might face.” Regarding these decisions, one shift head states that:

The negotiations have been very timely; I feel that at the beginning it cost a bit to decide to make changes, there was a point where we had to jump in and make big changes, like adding a shift, and the changes that had to be made were made. From then onwards, the efforts have been super timely and assertive, and they have made things easier for us, and for the team; they have been reassuring measures. (2)

The same email insisted on the use of N-95 masks in all areas of the unit, and the shift managers and all members of the unit were asked to supervise said indication. With regards to the communication and information channels used, the shift heads point out the following:

That the information is delivered is essential because it helps to reduce the anxiety that occurs in a context like this (...) Direct communication from the heads of shifts and heads of unit have stood out as a facilitating element to go through this period. (5)

What eases the situation is the direct conversation that we have had between the five shift heads and the supervisors; I know that I have a direct line to talk and find a solution. (3)

It’s calming that the communication channels have always been open. Although I may not have a particular piece of information here and now, I have the peace of mind that if I need that information, I can ask; and if someone on my team doesn’t know, my bosses will know; and if the bosses don’t know it, I am sure they will do everything possible to have the answer. (1)

Regarding infections of staff members, between April and July we had eight COVID-19 cases, of which at least three were infected outside of the hospital (Table 1); these numbers are low in comparison with the other units within the hospital. The head OB-GYN explains how the unit anticipated measures:

We have been ahead of the ministerial measures. Mainly the team of midwives and the coordinators and supervisor took the situation very seriously, and the prevention measures began much earlier than in other institutions. We started wearing a mask on a mandatory basis before the Ministry of Health said to do so. (8)

**TABLE 1:** The maternity unit’s health personnel in preventive quarantine and who tested positive for COVID-19 between April and July 2020; n = 103.

	COVID-19	Preventive quarantine
April	1	19
May	3	2
June	2	4
July	2	1
Total	8	26

Based on data from the Centro de Atención Integral del Funcionario (CAIF), Hospital Dra. Eloísa Díaz.

### The Path From the Suspension to the Reestablishment of Standards of Care

The measure of suspending the companion for the woman during labor and birth on March 22 hit the teams hard. Two of the shift heads report:

I feel like there is no valid justification to tell a woman she cannot have a companion; it’s preaching on something that you deeply disagree with. I don't know how to put it into words, it was just very terrible to have to do it. (1)

It was terrible because we were used to having our women being always accompanied by their loved ones during labor, delivery and their recovery. And at first, when they couldn’t have this accompaniment, it was very distressing, we felt very sad because they were not accompanied. (5)

It is interesting to note the team’s acknowledgment that fear played a central role in decision-making during the beginning of the pandemic. One of the shift head’s expresses:

We abused a bit of fear, and of the lack of knowledge, which led us to take exaggerated measures which perhaps were not so necessary and could have been avoided. (3)

Feelings of disapproval of the measures were expressed in the shift heads’ WhatsApp group during the last days of March, which led to discussing the feasibility of developing a protocol to admit companions again. The opinions were unanimous, and on March 31, the first draft of the protocol was presented, which was finally put into operation on April 9. The unit’s coordinator expresses:

They looked for evidence, they worked in coordination and support with the neonatal unit, they reviewed the best recommendations available. (...) The hospital gave us the support and gave us all the supplies we needed to be able to comply with the protocol. It was approved in record time. It was analyzed by the midwifery and medical team, by all heads, by the neonatology unit. It went through the different areas so that everyone could give their opinion on what was better and what to incorporate. (...) It was quite positive, and it happened mainly by the impulse and desire of the team, in an aim to be always respecting women's rights. (6)

One head of shift adds:

The team advocated for the rights of families, and on the other hand, the supervisors and heads were available to listen and see what resources were needed to make this possible. (3)

This protocol also addressed skin-to-skin contact in cases of suspected or positive COVID-19 women. The third version of the Chilean Pediatric Society’s protocol (from April 2) was used as a reference; it recommended skin-to-skin contact and breastfeeding in suspected or confirmed cases, unless the newborn was premature and/or the woman's illness condition didn’t make these possible. The companion during birth and skin-to-skin contact and breastfeeding were reestablished. Nevertheless, the midwives report on other negative impacts that the pandemic has had in the quality of care given, such as the effects of the use of PPE, as one midwife points out:

There is a physical issue; we are caring for women fully covered. We are putting a physical barrier of gloves, masks, hats, plastic. It is difficult to work as we know [that supporting] a woman in labor requires touch. You know you can support her in ways that are difficult to achieve through these plastic barriers. This might sound symbolic or even poetic, but it is certainly not the same. (1)

### When the Community Is Not Allowed in the Hospital, the Hospital Goes to the Community via Cellphones

The most usual form of initial contact of the Maternity with the community is through a guided tour through its premises that pregnant women and their couples take around their 36th week of pregnancy. Pre-pandemic, this tour was held every Monday, Wednesday and Friday at 11 AM: a midwife showed the facilities and explained the process of admission and care during childbirth. As a result of the pandemic, these tours were suspended, and the health team came up with the idea of conducting a “virtual guided tour” through the hospital's Instagram account. On April 22, the first guided Instagram tour was held, with great success: more than 100 people connected. The awareness of the uncertainty that the pandemic scenario generated for pregnant women led the team to request authorization to create an independent Instagram account for the entire Maternity. The medical residents’ head notes: “The creativity of the midwifery team has been amazing. The virtual guided tour has distinguished us as a Maternity” (7).

On April 29, the hospital management authorized the creation of the Maternity’s Instagram account, acknowledging that the initiative had been very well received in the external community, and also within the health team. A shift head points out:

A great milestone was the creation of the Instagram account. We have been able to contact the community from wherever we are, we have carried out many activities: lives, posts, answer questions and comments, connect the maternity with primary health care, keep in touch with women after they have left the Maternity. In addition, we get more direct and continuous feedback from our patients than before, which is crucial to improve our work. (2)

On May 15, the first Instagram publication was made, and content related to the World Respected Childbirth Week began to be uploaded. In addition to a first cycle of Instagram lives around that celebration, three other cycles have been held (Perinatal Dialogues, World Breastfeeding Week, and Dialogues in Midwifery). When we were completing the first draft of this article on August 31 2020, the account @maternidaddraeloisadiaz had more than 3,240 followers, of which 92% were women and 8% men. 56% of those who followed the account were 24–35 years old, followed by the 18–24 age group. One of the midwives in charge of the project declares:

I was motivated to create links and networks with users and with other national and international health institutions, and to be able to better connect within our own South-East Service network with other hospitals and primary health care. (I.3)

A team of six volunteer midwives answer the questions of the community 24/7. Regarding their motivations, they express: “I feel that being in direct contact with the users or with their relatives makes us closer to people” (I.1); “Our aim is to reduce anxiety in pregnant women, prevent them from going out, avoid unnecessary exposure to contagion” (I.2); “As the health system is organized in such a hierarchical way, it was difficult to achieve this closeness with the population through traditional channels” (I.3); “It interested me as a channel of information, and updated information, as a way to promote changes” (I.4). This team of volunteer midwives points out the importance and positive impact that the initiative has had:

It is super important to lower their anxiety and make them feel safe about the place where they are going to give birth; to show them that we are caring human beings, that there are no ranks or distinctions between us and them. (I.1)

Women express that they feel much calmer, they know that they have an open channel, that they can communicate with us in an expeditious way, they know that we answer at night, even very late. (I.2)

It makes the hospital closer to the community, it has calmed anxieties regarding COVID impressively, it has kept them much more informed. It has served to build affection toward the Maternity; they express they love the team, they love the way we work. (I.4)

Since the opening of the Instagram account on May 4 and until July 31, a total of 53 publications and 17 lives were done. In the same period, midwives answered the doubts of 228 Instagram users, in 434 interactions (dialogues of two or more messages between a user and a volunteer midwife). 77% of the interactions occurred with women from La Florida. Thus, on average, during our Instagram account’s first 88 days of operation, there were 4.9 daily interactions with users, of which 3.8 were with women from La Florida (Table 2). Of the total number of interactions during this period, 86% corresponded to questions, and 11% to congratulations for the usefulness of the maternity’s Instagram and for its work during the COVID outbreak, such as “I want to congratulate you for the page and for the information you share,” posted on May 29, and “Thanks for your Instagram lives! I feel empowered and informed for my birth at my 39 weeks of pregnancy … virtual hugs!” posted on August 14. Regarding birth experiences in the Maternity, on May 31, a new mother wrote:

Thank you for everything. Thank you for accompanying me in this beautiful moment. Despite everything that is happening, we felt cared for as if nothing wrong was going on, we received the best care imaginable. I will never stop thanking all the support and concern for me and for my daughter.

On July 30, another woman who had recently given birth in the Maternity commented:

Thank you very much for the tremendous work you do in the Maternity. Thanks to the midwife, to her words and her care, our daughter was born. As a couple, we saw most of the Instagram lives that the Maternity organized and they really helped us a lot to prepare for the birth. Thank you for the dedication, passion and love that you give in every birth and new life.

**TABLE 2:** Direct messages received on the instagram account @maternidaddraeloisadiaz between May 4 and July 31, 2020.

Variable	Number	%
Number of users	228	100
La Florida	150	65.7
Other	43	18.8
Undetermined	35	15.3
Number of interactions	434	
La Florida	335	77.1
Other	61	14
Undetermined	29	6.68
NA	9	2.07
Type of interaction		
Inquiries	374	86.1
Inquiry RPO/TDP	53	
Other	321	
Congratulations	46	10.5
Complaints	5	1.1
NA	9	2.0
Average of interactions per user (all users)	1.9	
Average of interactions per user (La Florida)	2.23	
Average of daily interactions (all users)	4.9	
Average of daily interactions (La Florida)	3.8	
Responses		
Yes	423	97.5
No	11	2.5
Resolution of inter-consultations	5	1.1

### The Maternity’s Standards of Care During the COVID-19 Outbreak

We have described our decision-making process as a maternity team since the beginning of the COVID-19 pandemic in Chile, until July 31, 2020. The first COVID-19 women were admitted to our Maternity in May 2020, after we had implemented protocols to protect the pre-pandemic standards of care. Even though the companion during birth was suspended during the last 10 days of March and first 10 days of April (a period in which we had no COVID-19^+^ women in labor), this decision had a big impact on our indicators: the 90% companionship during labor and birth (2019) dropped to 81.4% between January and July 2020—and even lower, to 70%, between March and April. Another figure that stands out is the difference between cesarean births, which were 27.8% of the total births in 2019, and 32.5% during the first 7 months of 2020 (Table 3). The head OB-GYN of the maternity explains this increase in cesareans:

We will have to look back at our practices. For example, cesarean sections, how it has increased, not only in our institution but in all hospitals in the Metropolitan Region, because we relaxed. We said: “People are stressed, we are not going to get so fussy about why we are conducting surgeries, we are not going to do surveillance, we are not going to audit,” and that has had the impact of an increase in our cesarean section rate of 7 points. It has affected a lot. (8)

**TABLE 3:** Main obstetric outcomes of all births in the maternity during 2019 and 2020 (until July 31st).

	2020 January–July)		2019 (January–December)	
	N	%	N	%
Total	1,518	100	3,003	100
Vaginal births	939	60.9	1975	65.7
Forceps	81	5.33	192	6.39
Cesareans	494	32.5	836	27.8
Skin to skin contact for >30′	1,064	70.9	2110	70%
Early breastfeeding	571	37.6	1,197	39.8
Companion during labor and delivery	1,236	81.4	2717	90.4
Apgar <7 at 5′	14	0.9	22	0.7

Based on monthly reports of OB/GYN Unit.

Between May and July 2020, 620 births occurred in our maternity. Fifty five (8.9%) of these women tested positive for RT-PCR SARS-CoV-2 (52/55 delivered within 14 days after diagnosis of COVID-19). Demographic features, clinical characteristics and delivery outcome of COVID-19 cases are shown in Table 4. Twenty one cases were migrant (39%) and 45 (82%) were residents of La Florida. Two COVID-19^+^ women had respiratory comorbidities (asthma). Fifty four infections were diagnosed in the third trimester, 41 (75%) were asymptomatic; two were admitted to IMCU (non-invasive respiratory therapy) and two were admitted to the ICU.

**TABLE 4:** Demographic features, clinical characteristics and delivery outcomes of COVID-19^+^ women with RT-PCR (n = 55).

	(n) ^/a^ mean	Percentage [interval]
Nationality		
Chile	34	61%
Venezuela	8	14%
Haiti	6	11%
Colombia	2	4%
Peru	2	4%
Other: Argentina, Bolivia and Dominican Republic.	3	6%
Residence (municipality)		
La Florida	45	82%
Puente Alto	3	5%
La Granja	2	4%
La Pintana	2	4%
Other municipalities (3)	3	5%
Maternal age (years)	29.4^/a^	[15–41]
Parity		
0	21	38%
1	19	35%
2	10	18%
3 and more	5	9%
Pregnancy trimester at diagnosis		
First	0	—
Second	1	25^+0^
Third	54	[28^+1^–41^+0^]
Respiratory comorbidities	2	3.6%
Symptoms		
No	41	75%
Fever	3	6%
Cough	4	7%
Dyspnea	5	9%
Other	3	6%
Route of delivery		
Vaginal	36	65.4%
C-section	15	27.3%
C-section (maternal health condition) COVID-19	4	7.3%
ICU Admission	2	3.6%
IMCU admission	2	3.6%
Gestational age at delivery (weeks)		
Extreme preterm (earlier than 28 weeks of gestation)	1	1.8%
Early preterm (earlier than 34 weeks of gestation)	2	3.6%
Late preterm (34–36 weeks of gestation)	6	10.9%
Early term (37–38 weeks of gestation)	18	32.7%
Full term (39–40 weeks of gestation)	25	45.5%
Late term (41 weeks of gestation)	3	5.5%
Post term (42 weeks of gestation and beyond)	0	—
Pregnant women who delivered within 14 days after diagnosis of COVID-19 (RT-PCR)	52	94.5%

All women with severe COVID-19 (four cases) had a preterm delivery by cesarean and the indication was related to COVID-19 infection. Thirty six women (65.4%) with non-severe symptoms had a vaginal delivery and 15 had a cesarean birth for indications non-related to COVID-19. Although these 36 cesareans were indicated as “nonrelated” to COVID-19, we can say that the pandemic context had an impact on the criteria to perform these surgeries, as, during the pandemic, the staff became less rigorous in applying the Maternity’s cesarean protocols, and in case of doubt on the physiologic progress of labor, they preferred to practice an emergency cesarean, as that provided better opportunity for the proper use of PPE.

Of the total number of 55 women with COVID-19, 29 (52.7%) were under conditions of isolation, and therefore in one of the Comprehensive Childbirth Rooms, with all the security measures established for these cases. The remaining 26 women were treated “blindly”—that is, in ignorance of the result of the RT-PCR, which began to be taken routinely on May 15th on all pregnant women who were admitted into the Maternity. Processing the exam took between 48 and 72 h, which is why in many cases, the diagnosis was only learned in the postpartum period, and therefore these women were not treated with the strictest isolation measures in a room equipped for that. The only option to enter the isolation unit was to have a known RT-PCR(+), to present symptoms, or to have referred as a COVID-19 contact. In this group, the standards that were offered can be seen in Table 5. Fifteen women had companions, and it stands out that from the total of 14 vaginal deliveries, of which 12 (86%) were assisted by a midwife, the skin-to-skin contact after birth for at least 30 min (which is the minimum time for contact recommended in the national protocols) reached 86%, well above the average in 2020 (70%). (Often this skin-to-skin contact lasted much longer.) The latter is accounted for by the efforts made by the health teams to promote birth rights even in these cases.

**TABLE 5:** Clinical characteristics, delivery outcomes, and standards of care of COVID-19 positive women with RT-PCR who delivered under isolation conditions (n = 29).

	Vaginal n = 14 48%	C-section n = 11 38%	C-section COVID-19 indication n = 4 14%
Maternal age (years; mean, range)	31.1 [18–40]	29.4 [22–41]	33.3 [30–36]
Symptoms (number; percentage)	2 (14%)	1 (9%)	4 (100%)
Gestational age at delivery			
Early preterm (<34 weeks)	0 (0%)	0 (0%)	2 (50%)
Late preterm (34–36 weeks)	1 (7%)	0 (0%)	2 (50%)
Term (>37 weeks)	13 (93%)	11 (100%)	0 (0%)
ICU Admission	0	0	2 (50%)
Birth weight (grams; mean, range)	3,144 [2,710 – 3,720]	3,032 [2,180 – 3,480]	2,291 [1,760 – 3,245]
Apgar score 5 min > 7	14 (100%)	11 (100%)	2 (50%)
Pregnant woman accompanied during delivery (by her partner or trusted support person)	11 (79%)	4 (36%)	0 (0%)
Skin-to-skin contact (30 min or more)	12 (86%)	3 (27%)	0 (0%)
Birth attended by midwife	12 (86%)	0 (0%)	0 (0%)

## Discussion

In this article, we have sought to describe the responses to the COVID-19 pandemic in the Eloísa Díaz public Maternity. As we have shown, this Maternity unit already stood out for its high standards of care since before this health crisis, implementing a humanistic model of childbirth care as described by Davis-Floyd (2001), Davis-Floyd (2018), and was able to maintain almost the same standards during the first months of the COVID outbreak (March to July).

In the La Florida Maternity, during 2019, 70% of our clients had immediate skin-to-skin contact of 30 min or more after birth, including breastfeeding, and during 2020 (until the end of July), it was 71%. Ninety percent of labors and births had a companion in 2019, which decreased to 81% in the same period of 2020 (Table 3). But, as we have shown, this fall reflects the ban on companionship that occurred for only 20 days, during which the Maternity’s midwives pressed for protocols to reverse the measure. Once the proposals to reestablish previous standards of care were shared, all heads were supportive. This shows the strength with which a collective culture of respect for the rights of women and newborns has been instilled in the Maternity staff, as well as that of non-compliance with measures that do not have supporting evidence. As one of the midwives’ head of shift expresses: “There were certain standards of care that we wouldn’t compromise, such as the suspension of skin-to-skin, of a companion during birth; we were not just going to normalize it because someone told you it should be done or it ‘seemed’ to be adequate” (3).

One of our health team’s fear was that the rate of cesarean births would rise during the pandemic, as had been reported in several countries and contexts by April. A systematic review that included 108 births of women diagnosed with COVID-19 showed that 91% had a cesarean birth (Zaigham and Andersson, 2020). A study in Spain with 82 COVID-19^+^ women showed a rate of 47% of cesareans (Martínez-Perez et al., 2020). In June, the findings from a rapid online global survey of maternal and newborn health professionals facing the COVID-19 pandemic, which included responses from 81 countries, showed that cesareans were commonly performed among women diagnosed with COVID-19, and some facilities aimed to reduce labor duration and time spent in the labor room by performing cesareans even on non-infected women (Semaan et al., 2020). In our Maternity, in contrast, the cesarean birth rate was 34.6% in the group of 55 women with COVID-19, 6.8% higher than during 2019 (Tables 3 and 4). Although this is an increase from our pre-pandemic standards, it is still lower than most public maternities in the country, which had a cesarean average of 41% in 2015 (National Institute of Human Rights, 2016); and the maternity’s personnel has acknowledged the increase and is working on ways to reverse it.

The restrictions on in-person visits to the maternity and difficulty in accessing updated information on maternity protocols contributed to an increase in the anxiety that pregnant women and their families were already living with due to the pandemic, which motivated us to look for new ways of contact with them. As an editorial published in *Women & Birth* (Matvienko-Sikar et al., 2020) acknowledges, the COVID-19 pandemic has created the context for increased experiences of distress related to: restrictions in normal routines; concerns about the risk of infection; changes in antenatal care and in access to perinatal health care; restrictions on the presence of partners during maternity care; as well as reduced access to support networks. In this scenario, support from networks and from midwives and other healthcare professionals is critical for women's mental health during the pandemic. In our Maternity, the awareness of this increased mental health vulnerability of our community led us to the creation of an Instagram account as a communication channel and to carry out “virtual visits” to our premises. This channel was managed until the end of July by six volunteer midwives; during August, their number grew to 10. These volunteers continue to interact with the community on an ongoing basis, and we already anticipate that this communication channel must be strengthened and has great future, post-pandemic potential.

Another relevant element we must include is the creation, on July 23, of the Dra. Eloísa Díaz’s Hospital “Gender Roundtable,” which has begun an awareness campaign to reject any practice or conduct that violates women's rights within the hospital (Dra. Eloísa Díaz Hospital, 2020). Among the members of this unprecedented initiative are a midwife (one of our Instagram volunteers) and a woman ob-gyn from our Maternity unit.

The great awareness of women’s and communities’ rights to dignified healthcare has been facilitated by our staff of midwives, who are predominantly young (mostly in their 30s) and who have actively served in the social movement for greater equality and dignity for all that has mobilized Chile during the last 15 years. Many of our team’s members were part of the student movement referred to as the “Penguin Revolution” of 2006, which demanded quality and free education, and of the massive social uprising of October 2019 against inequality and the unresolved needs of the population for improved education, pensions and health, as one midwife clearly expresses:

The fact that our Maternity has young health teams right now was very, very beneficial for us. We are a generation that has faced many changes, and that has acted as a protective factor for us: the fact that we can adapt, that we are used to change, that we are a generation of changes, that we have demanded changes in our country. We are used to it, we were the generations of the “penguins,” we were part of the student strikes, we have been part of the October revolt. This means we believe in our capacity to change things—not drown in problems but rather to face them and search, participate, engage, try to find creative solutions … That is our greatest strength and what differentiates us from other institutions. (3)

Furthermore, the sense of a collective purpose has made the team grow closer and stronger. In the words of a head of shift: “This has made us become more family than we already were” (3). The OB-GYN head of residents acknowledges:

These emergency situations consolidate work teams. Despite all the things that can happen on a daily basis, all of us have experienced the same in our working context, and we have all tried to work for the same cause. I think that is the most important thing. I have always said that we are a team, that we cannot work without midwives and technicians, and that was shown in this pandemic, that we are a team. (7)

With this devoted young team, with ease to adapt to change, with a profound gender and human rights approach that has been able to protect women and newborns’ rights during the COVID-19 pandemic, we hope to lead the way for other maternities in the country and region to follow similar paths, and we are expecting to join the *International Childbirth Initiative (ICI): 12 Steps to Safe and Respectful Maternity Care* in the near future (Lalonde et al., 2019; www.internationalchildbirth.com).

Finally, an issue of great concern today is the maternity staff’s mental health, which has been stressed to the maximum in the context of COVID-19, as our heads of shift express: “The whole team is subjected to a higher basal stress, and that has had repercussions especially on mental health, which will increase in the near future” (2); “An emotional drain, I think that is by far what is going to weigh the most. The stress of being in a situation in which you may not want to be, I mean the stress that everything is based on fear” (3). The concern for mental health is consistent with the preliminary results of the study “The impact of the COVID-19 pandemic on the mental health of workers in health services” in the country: of the 954 health workers interviewed throughout the country, 37.6% report lack of energy and fatigue, 38.6% lack of appetite and, most worryingly, 31.4% present moderate to high depressive symptoms (Medical College of Chile, 2020).

## Data Availability

The raw data supporting the conclusions of this article will be made available by the authors, without undue reservation.

## Author's Note

A Spanish translation of this article is available at Supplementary Material.
