# Supplementary material for: Protecting Women’s and Newborns’ Rights in a Public Maternity Unit During the COVID-19 Outbreak: The Case of Dra. Eloísa Díaz - La Florida Hospital in Santiago, Chile
Source: Front Sociol. 2021 Mar 2;6:614021. doi: 10.3389/fsoc.2021.614021 (PMC8022682; doi:10.3389/fsoc.2021.614021)
Supplement: Supplementary file 1 [file DataSheet1.pdf]

OPEN ACCESS

Editado por:
Robbie Elizabeth Davis-Floyd, Rice University, United States

Revisión de pares:
Mounia El Kotni, École des Hautes Études en Sciences Sociales, France
Pamela Downe, University of Saskatchewan, Canada

Correspondencia:
Gonzalo Leiva
gonzalo.leiva.rojas@gmail.com

†ORCID:
Gonzalo Leiva http://orcid.org/0000-0002-0654-2615
Michelle Sadler http://orcid.org/0000-0002-3895-047X
Camila López http://orcid.org/0000-0002-4718-1588
Susana Quezada http://orcid.org/0000-0002-7532-8794
Víctor Flores http://orcid.org/0000-0003-2298-8614
Christian Sierra http://orcid.org/0000-0002-3737-4132
Susan Díaz http://orcid.org/0000-0002-2236-5647
Christian Figueroa http://orcid.org/0000-0001-94468568

Specialty section:
This article was submitted to Gender, Sex and Sexualities, a section of the journal Frontiers in Sociology

Recibido: *05 Octubre 2020*
Aceptado: *15 Enero 2021*
Publicado: *02 Marzo 2021*

Citación:
Leiva G, Sadler M, López C, Quezada S, Flores V, Sierra C, Díaz S and Figueroa C (2021) Protecting Women's and Newborns' Rights in a Public Maternity Unit During the COVID-19 Outbreak: The Case of Dra. Eloísa Díaz - La Florida Hospital in Santiago, Chile. Front. Sociol. 6:614021. doi: 10.3389/fsoc.2021.614021

Protegiendo los derechos de la mujeres y recién nacidos en una maternidad pública durante el brote de COVID-19: el caso del Hospital Dra. Eloísa Díaz – La Florida, en Santiago, Chile.

Gonzalo Leiva[1,2,3*†], Michelle Sadler[3,4†], Camila López[1†], Susana Quezada[5†], Víctor Flores[1†], Cristian Sierra[1†], Susan Díaz[1†] and Christian Figueroa[1,2†]

[1]Maternidad Hospital Dra. Eloísa Díaz-La Florida, Santiago, Chile, [2]Facultad de Medicina, Pontificia Universidad Católica de Chile, Santiago, Chile, [3]Observatorio de Violencia Obstétrica, Santiago, Chile, [4]Facultad de Artes Liberales, Universidad Adolfo Ibáñez, Santiago, Chile, [5]Consultora Internacional sobre políticas y programas de desarrollo infantil temprano, y de salud materna infantil, Washington, DC, United States.

La maternidad del Hospital Dra. Eloísa Díaz, ubicada en la comuna de La Florida en la ciudad de Santiago de Chile, abrió sus puertas en 2014, y ha integrado un modelo humanista de atención denominado "Modelo de Parto Personalizado Seguro" desde el año 2016. Con alrededor de 3.000 nacimientos por año, ha sido reconocida como un ejemplo de excelencia en atención de maternidad en el país. El brote de COVID-19 presentó un gran desafío para esta maternidad: mantener sus estándares de calidad de atención a pesar de la crisis de salud. Este artículo presenta las respuestas de la maternidad a la pandemia entre marzo y julio de 2020, describiendo las estrategias que se implementaron y sus resultados obstétricos. Se llevaron a cabo entrevistas semiestructuradas a matronas(es) y médicos(as) ginecobstetras, y una revisión retrospectiva de los estándares de atención al parto y de los resultados perinatales de las 55 mujeres que dieron positivo a SARS-CoV-2. Los resultados muestran cómo el personal de la maternidad respondió para evitar un impacto negativo significativo en los derechos de las mujeres y los recién nacidos. Protocolos para restablecer el acompañamiento durante el trabajo de parto y parto y el contacto piel con piel, que habían sido suspendidos por casi tres semanas al inicio del brote, así como la creación de una cuenta de Instagram para comunicarse con la comunidad, fueron algunas de las medidas tomadas. Después de algunas semanas iniciales de ajuste, los estándares de atención para todas las mujeres, incluidas las diagnosticadas con COVID-19, fueron restablecidos casi a niveles pre pandémicos. Este caso muestra que se puede mantener la calidad de la atención y se pueden respetar los derechos de las mujeres y los recién nacidos durante una crisis de salud como la pandemia de COVID-19.

Palabras clave: parto humanista, derechos humanos en el parto, COVID-19, salud pública, matronería, partería.

INTRODUCCIÓN

Chile y su sistema de salud

Chile es una República democrática localizada en América del Sur, con una población de 17,574,003 habitantes en 2017 (National Institute of Statistics, 2018). Es considerado un país de altos ingresos con un Producto Interno Bruto (PIB) per cápita de US$ 25,041 en 2019 (World Bank, 2020). Durante 2017, 8.6% de la población vivía en pobreza y 0.3% vivía con menos de $1.90 USD por día (Ministry of Social Development and Family, 2018; World Bank, 2020). Sin embargo, mas del 30% de la población es económicamente vulnerable y la inequidad de ingresos económicos permanece alta (World Bank, 2020). El sistema de salud chileno ha mantenido una estructura que fue definida durante los años 80 (ECLAC, 2012), la que consiste en un sector privado y uno público tanto para el aseguramiento como para la provisión de servicios, que se financian a través de contribuciones sociales, impuestos generales, co-pagos y aportes voluntarios. El total de gasto en salud per cápita fue de US$2,159 en el año 2019, representando el 9.1% del PIB del país. El gasto público representa el 5.4% del PIB y el gasto de bolsillo alcanza el 33.9% del gasto en salud (OECD, 2020). La provisión de servicios públicos de salud es altamente descentralizada y coordinada por el Sistema Nacional de Servicios de Salud, el que consiste en 29 Servicios de Salud distribuidos geográficamente. El aseguramiento de la salud es provisto a través de proveedores privados (ISAPREs) y en mayor medida por el Fondo Nacional de Salud (FONASA), que cubría al 78% de la población en el año 2017. El sector público, a diferencia del privado, se caracteriza por déficits de infraestructura hospitalaria, baja privacidad en la atención al paciente, impersonalidad en el tratamiento de los pacientes, condiciones laborales desfavorables y bajos niveles salariales (Goic, 2015). El 92% de quienes pertenecen al decil de ingresos más bajos de la población y el 80.6% de las mujeres son beneficiarios del seguro público de salud (Ministry of Social Development and Family, 2018). Los beneficiarios de FONASA tienen acceso a dos modalidades de planes de salud, lo que es determinado por el ingreso del hogar y el número de integrantes de este: modalidad de atención institucional (MAI) y modalidad de libre elección (MLE). Respecto de la atención del parto, la MAI no requiere pago de bolsillo, a diferencia de la MLE, que implica un gasto de bolsillo de alrededor de US$360 en septiembre de 2020 (National Public Health Insurance Fund, 2020).

En 2017 se registraron 219,186 nacidos vivos en el país; y la cobertura de parto asistido por profesionales alcanzó el 99.7% (National Institute of Statistics, 2019); la tasa de mortalidad infantil (muertes/1,000 nacidos vivos) fue de 6/1,000 (UNICEF, 2018); y la mortalidad materna (muertes maternas/100,000 nacidos vivos) fue de 13/100,000 (WHO, 2019). Si bien son buenos indicadores de salud materna, son cifras que esconden grandes brechas en el acceso y la calidad de la atención de salud entre los establecimientos de salud públicos y privados, disparidades entre territorios y falta de opciones con respecto al lugar de ocurrencia del nacimiento. Los seguros de salud solo cubren los partos en instalaciones hospitalarias, y el sistema de salud desaconseja y no reconoce el parto en el hogar, aunque este no es ilegal.

Más de ¾ de los nacimientos del país, 77.4%, ocurrieron en el sector público en el año 2017 (Ministry of Health, 2020a). Aunque se ha reportado que la calidad de la atención interpersonal entre el personal de salud y las mujeres es mejor en el sector privado que en el público (OVO Chile, 2018), ambos sectores de la salud exhiben intervenciones obstétricas de rutina alarmantemente altas durante el parto, como lo indica la tasa nacional de cesárea que alcanzó un 50% en 2015 (National Institute of Human Rights, 2016). Un estudio realizado en nueve importantes maternidades de hospitales regionales públicos, en el que participaron mujeres primíparas y multíparas admitidas en trabajo de parto, reportó las siguientes intervenciones: 91% tuvo trabajo de parto inducido médicamente, 55% tuvo monitoreo intraparto fetal continuo, 56% tuvo episiotomías y 80% tuvo su parto en posición de litotomía (Binfa et al., 2016). La tasa de intervenciones obstétricas es igualmente alta en la salud privada (OVO Chile, 2018), siendo las cesáreas un 27% más altas en el sector privado que en el público (National Institute of Human Rights, 2016).

Chile es reconocido internacionalmente por un programa para el desarrollo de la primera infancia llamado "Chile Crece Contigo" (Richter et al., 2016), que tiene cobertura nacional desde 2008. Un componente eje de esta política intersectorial es el Programa de Apoyo al Desarrollo Biopsicosocial (PADB), que, entre otros, tiene como objetivo fortalecer la atención prenatal y brindar atención personalizada durante el parto. (Ministry of Social Development and Family and World Bank, 2018). De la mano de este programa, en 2008, el Ministerio de Salud lanzó el Manual de Atención Personalizada en el Proceso Reproductivo (Ministry of Health, 2008), con el objetivo de implementar un modelo humanista de atención al nacimiento (Davis-Floyd, 2001; Davis-Floyd, 2018). Si bien estas políticas y recomendaciones han ayudado a mejorar algunas prácticas, principalmente aquellas que constituyen indicadores de PADB (como la presencia de un acompañante durante el trabajo de parto y el parto, y el contacto piel a piel con el recién nacido durante 30 o más minutos inmediatamente después del nacimiento), no ha habido un cambio profundo de paradigma a nivel nacional hacia la humanización del cuidado durante el trabajo de parto y el parto. Así, los esfuerzos del sector salud para mejorar la atención de la maternidad durante la última década han tenido un impacto limitado (Binfa et al., 2016; OVO Chile, 2018).

El SARS-CoV-2 en Chile y su amenaza al cuidado materno respetuoso y los derechos de nacimiento

En Chile se notificó el primer caso de COVID-19 el 2 de marzo de 2020. Exactamente dos semanas después, el 16 de marzo, el Ministerio de Salud señaló que Chile estaba entrando en la Fase 4, que corresponde a la etapa de propagación comunitaria sostenida del virus. Ese mismo día, las escuelas suspendieron sus actividades en todo el territorio nacional, y dos municipios de la Región Metropolitana declararon el estado de emergencia: Santiago y La Florida (Municipality of La Florida, 2020). Esta decisión se tomó dos días antes de la publicación oficial del Decreto Supremo No. 104, que declaró estado de excepción constitucional de catástrofe, por calamidad pública, en el territorio de Chile (Ministry of the Interior and Public Security,

2020). El 22 de marzo se inició un toque de queda en todo el país, para que la población permaneciera en el interior de sus casas, desde las 22:00 a las 05:00 horas. Y, desde el 26 de marzo, se implementaron cuarentenas “dinámicas”, definiendo medidas más duras o blandas, en varias regiones, de acuerdo con sus indicadores relacionados con COVID-19. Esto ha significado abrir algunas comunidades mientras otras permanecen cerradas, o viceversa, incluso cuando se trata de territorios vecinos.

El municipio de La Florida es el cuarto más grande de la Región Metropolitana; cuenta con una población de 402,433 habitantes que representa el 5% de los habitantes de dicha región (Ministry of Health, 2020b). Este municipio entró en cuarentena el 15 de mayo y permaneció en ese estado hasta el 30 de agosto de 2020, fecha en que enviamos este artículo por primera vez.

El Informe Epidemiológico N° 39, publicado por el Departamento de Epidemiología (Ministry of Health, 2020b), informó que el 2 de agosto (5 meses después del primer caso reportado), 401,142 personas habían sido infectadas y 9,968 habían muerto por COVID-19 en el país. Esto colocó a Chile en la octava posición de casos diagnosticados en el mundo en esa fecha (WHO, 2020a); y como el tercer país con mayor tasa de mortalidad por millón de habitantes, con 53,17 muertes por millón, solo después del Reino Unido (70,08) y Perú (64,55) (John Hopkins University, 2020).

No había duda de que COVID-19 supondría un desafío para los sistemas de salud de todo el mundo, pero no estaba claro cuál sería su impacto específico en la atención de maternidad. La "Guía de atención de mujeres embarazadas con sospecha de infección por SARS-CoV-2" (Favre et al., 2020), publicada el 3 de marzo, sugirió acortar el trabajo de parto para evitar el agotamiento en mujeres con infección confirmada. Aunque indicaron que no había evidencia de transmisión vertical del virus, los autores recomendaron el aislamiento del recién nacido durante al menos 14 días o hasta que desapareciera la propagación viral, tiempo durante el cual no se recomendaba la lactancia materna (Favre et al., 2020). Esta guía, que podría acarrear “consecuencias de magnitud impredecible a largo plazo” según los autores, fueron traducidas al español (Martinez-Portilla et al., 2020) y ampliamente difundidas, a pesar de la evidencia demostrando el daño que esas medidas podrían causar (Schmid et al., 2020; Smith, 2020; Stuebe, 2020).

Hasta mayo, según una revisión de 15 artículos publicados por 10 sociedades científicas, incluida la OMS, no había evidencia definitiva que sugiriera la transmisión vertical del SARS-CoV-2. Asimismo, esta revisión concluyó que no es aconsejable separar a las madres de los recién nacidos ni desalentar la lactancia, a menos que la madre esté gravemente enferma (Narang et al., 2020). Pero a pesar de la evidencia y del hecho de que la OMS (2020b) había señalado desde principios de marzo que las mujeres embarazadas deberían tener acceso a una atención especializada, respetuosa y centrada en la mujer, a medida que se propagaba la pandemia, las voces de alerta en todo el mundo expresaban preocupación por los derechos de las mujeres y los recién nacidos durante el parto (International Confederation of Midwives, 2020; Human Rights and Childbirth, 2020; Sadler et al., 2020). Organizaciones no gubernamentales denunciaron que en muchos lugares del mundo, a las mujeres se les estaba: acelerando innecesariamente el trabajo de parto (inducción programada del trabajo de parto, oxitocina de rutina, partos instrumentales); indicando cesáreas programadas como única opción de vía de parto; negando el acompañamiento durante el trabajo de parto y el parto; separando rutinariamente de sus recién nacidos; y desaconsejando o prohibiendo la lactancia (Childbirth is Ours, 2020; OVO Chile, 2020).

En nuestro país, la Sociedad Chilena de Pediatría publicó las primeras guías para madres con COVID-19 y sus recién nacidos a mediados de marzo. En estas, no se recomendaba el contacto piel a piel en mujeres con síntomas, independientemente de la gravedad de la enfermedad; se promovía el pinzamiento temprano del cordón; y no se recomendaba la lactancia materna para las madres COVID-19^{+}. En la tercera versión de este documento, publicada el 2 de abril, se revisaron algunas de estas medidas, recomendando el contacto piel con piel y la lactancia materna en madres confirmadas o sospechadas de COVID-19, siempre que sus bebés nacieran a término y que las madres no se encontraran en estado grave (Chilean Pediatric Society, 2020). El Ministerio de Salud tardó casi 4 meses desde el primer caso de COVID reportado en el país, en emitir lineamientos técnicos sobre la atención a la maternidad. Hasta el 25 de junio, cada Servicio de Salud y cada maternidad estaba respondiendo a la pandemia según sus propios recursos y decisiones locales. El Observatorio de Violencia Obstétrica de Chile informó que, a fines de mayo, el 46% de los hospitales públicos habían suspendido por completo el acompañamiento durante el trabajo de parto y el parto, situación que no ocurría en ninguna clínica privada del país (OVO Chile, 2020).

Hasta el 12 de junio, solo existían rumores de la existencia de documentos técnicos que no habían sido autorizados oficialmente. En esa fecha se expidió la Ordenanza N° 1891 sobre recomendaciones para la atención de puérperas, niños y niñas, respecto a factores de riesgos de morbimortalidad materno-infantil y su salud mental, en pandemia COVID-19, recomendando la presencia de un acompañante durante el parto con el adecuado equipo de protección personal (EPP), contacto piel a piel después del nacimiento y lactancia (Ministry of Health, 2020c). Fue recién el 25 de junio, luego de una fuerte presión de las organizaciones de la sociedad civil y del Colegio Nacional de Matronas (Chilean College of Midwives, 2020), que se publicaron las nuevas “Orientaciones para el manejo de casos SARS-CoV-2 (COVID-19) en gestantes, puérperas y/o díadas”. El documento recomienda el acompañamiento en los casos de mujeres con COVID-19, contacto piel a piel y lactancia, como lo había hecho anteriormente la Sociedad Chilena de Pediatría. En cuanto al alojamiento conjunto, recomienda tomar decisiones en función de cada realidad institucional (Ministry of Heath, 2020d).

Desde el inicio de la pandemia, la mayoría de las maternidades públicas en todo el país retrocedieron en los estándares de atención que habían ido mejorando gradualmente durante la última década. Pero algunos hicieron grandes esfuerzos para mantener la calidad de la atención que habían logrado. Este fue el caso de la maternidad del Hospital Dra. Eloísa Díaz, de La Florida, que opera desde fines de 2014 y que ha sido reconocido como un ejemplo de excelencia en la atención materna en el país (South-East Metropolitan Healthcare Service, 2020). Como breve contexto, la comuna de La Florida había reportado 13,357 casos de COVID-19^{+} al 2 de agosto (la cuarta comuna con más casos en la Región

Metropolitana) y 451 defunciones (segunda en la Región) (Ministry of Health, 2020b).

Había un gran desafío por delante, el mayor en la corta historia de dicha maternidad: enfrentar la mayor amenaza a la salud de la población del que teníamos registro, manteniendo los estándares de atención que habíamos alcanzado. Todo esto, en una de las comunas más grandes del país y en una de las más afectadas por la pandemia.

MÉTODOS

Abordamos el proceso de descripción y sistematización de la respuesta al brote de COVID-19 por parte de la maternidad del Hospital Dra. Eloísa Díaz, en el periodo entre marzo y julio de 2020, utilizando metodología mixta. Se realizaron entrevistas cualitativas semiestructuradas para indagar sobre las experiencias y percepciones del personal de salud con ocho funcionarios clave de la maternidad: seis matronas(es) y dos médicos(as) obstetras. De las(os) matronas(es), cinco eran jefas(es) de turno durante el período estudiado y una era la supervisora de todos los turnos. Una médico ginecobstetra era la jefa de residentes, y un médico era el jefe de obstetricia y ginecología de la maternidad. Todas las personas participantes firmaron un consentimiento, las entrevistas fueron realizadas por uno de los autores de este articulo a través de Zoom y fueron grabadas, con una duración promedio de 35 minutos. La guía de entrevistas cubrió los siguientes temas: impactos del SARS-CoV-2 en los funcionarios de la maternidad y a nivel personal; impactos en los estándares de atención; opiniones sobre el trabajo de la dirección y sobre la información y comunicación durante el brote; hitos o elementos clave identificados para mantener los estándares de atención previos; y recomendaciones para el trabajo futuro. Adicionalmente, cuatro de las matronas a cargo de la recién creada cuenta de Instagram de la maternidad, respondieron a una breve guía de entrevistas a través de llamadas por WhatsApp, sobre los siguientes temas: el proceso que llevó a la creación de la cuenta, la motivación para participar en el proyecto y el impacto de la cuenta en su trabajo y en los estándares de atención de la maternidad. De estas cuatro matronas, una también había respondido a la entrevista semiestructurada, ya que era jefa de turno y también una de las voluntarias de Instagram.

Todas las entrevistas fueron transcritas textualmente, codificadas y analizadas mediante análisis temático, un método cualitativo que permite identificar patrones temáticos a partir de los datos recopilados (Creswell, 2014). Los tres primeros autores transcribieron las entrevistas, leyeron y releyeron las transcripciones para asegurar la precisión de la grabación, y codificaron las entrevistas. Los códigos a priori que describen amplias dimensiones analíticas se derivaron de las guías de entrevista, y se desarrollaron códigos inductivos a medida que se examinaron los datos. Tras el proceso de codificación, los otros autores se unieron al siguiente proceso de interpretación, que se produjo de forma iterativa, revisando las interpretaciones y llegando a conclusiones. Al citar a nuestros interlocutores a lo largo de este artículo, seguiremos un sistema de numeración cronológico, con (1)-(8) haciendo referencia a las entrevistas semiestructuradas realizadas en ese orden; y a (I.1)-(I.4) al aludir a las cuatro breves entrevistas que describen la cuenta de Instagram.

A pesar de nuestra intención inicial era entrevistar a mujeres y familias que habían recibido atención dentro de la maternidad, privilegiamos las entrevistas con el personal de salud por dos principales razones: en primer lugar, para comprender en profundidad el proceso de toma de decisiones que se produjo en respuesta a la pandemia; y en segundo lugar, por la poca disponibilidad de tiempo para realizar la investigación, dado que la mayoría de los autores se encontraban trabajando como personal de salud en la maternidad durante el brote de COVID-19. Para incluir las experiencias de mujeres, el artículo contiene algunos testimonios de usuarias publicados en la cuenta de Instagram de la maternidad. Además, y con respecto al impacto de la cuenta de Instagram, realizamos un análisis cuantitativo de las interacciones en esa cuenta durante sus primeros 88 días de operación (del 4 de mayo al 31 de julio), detallando el número de usuarios e interacciones, y citando algunos de las publicaciones.

Para reportar los principales resultados obstétricos, recolectamos la información de todos los partos ocurridos en la maternidad durante 2019 y 2020 (hasta el 31 de julio) de las bases de datos del Hospital, y realizamos una revisión retrospectiva de 55 gestantes con confirmación por laboratorio de COVID-19 (isopado nasofaríngeo materno positivo para SARS-CoV-2) que ingresaron en la maternidad y dieron a luz entre el 1 de mayo de 2020 y el 31 de julio de 2020. Matronas(es) de la maternidad recopilaron los datos de los casos de COVID-19^{+} y los sistematizaron en una tabla de variables obstétricas a partir del conjunto de datos intraparto extraídos de la base de datos del American College of NurseMidwives (ACNM), que fue modificada y adaptada por los investigadores a la realidad local y al contexto de la pandemia. El protocolo de estudio fue aprobado por el Comité de Ética Científica del Hospital Dra. Eloísa Díaz.

RESULTADOS

Modelo de Parto Personalizado Seguro en el Hospital Dra. Eloísa Díaz

El Hospital Dra. Eloísa Díaz forma parte del Servicio de Salud Metropolitano del Sur-Oriente y es uno de los 198 hospitales de diferentes complejidades que conforman la red pública del país. El hospital entrega atención a las mujeres registradas en los nueve centros de atención primaria de La Florida. Entre 2016 y 2019, se registró un promedio de 4,470 nacidos vivos por año en La Florida (Ministry of Health, 2020e), de los cuales un promedio de 66% ocurrió en la maternidad del Hospital Dra. Eloísa Díaz. Entre enero y julio de 2020, el 70% de todos los nacimientos de la comuna ocurrió en esta maternidad (estimación realizada por los autores con base en los datos municipales), que atiende a las mujeres aseguradas bajo la Modalidad de Atención Institucional (MAI), la que no requiere un pago extra de bolsillo (además del de FONASA).

La unidad de ginecología y obstetricia, en la que se centra este artículo, es una de las tres unidades del Centro de Responsabilidad de la Mujer (en adelante CR de la mujer). La unidad tiene cuatro áreas: una sala de recuperación con seis

camas; cinco salas quirúrgicas para partos vaginales y cesáreas, así como para las cirugías obstétricas y ginecológicas electivas y de emergencia; una sala de preparto con ocho camas, donde solo tiene lugar el trabajo de parto (en la segunda etapa del trabajo de parto, las mujeres se trasladan a una sala quirúrgica); y cuatro salas integrales de parto dentro del área quirúrgica, para el preparto y parto vaginal. Las mujeres son ingresadas a la sala de preparación tradicional y a las salas gineco-obstétricas o a las salas integrales de parto, dependiendo de sus factores de riesgo y de la disponibilidad de salas. La unidad se organiza en dos tipos de equipos, denominados como equipo "médico" y equipo "no médico". El equipo médico está compuesto por 18 médicos ginecobstetras organizados en grupos de tres, que rotan cada seis días, y que están disponibles para las tres unidades del CR de la mujer (emergencia, ginecología y obstetricia y hospitalización). El equipo no médico está compuesto por 103 trabajadores de la salud. Del total del personal, 14 trabajan durante el día, de lunes a viernes, y el resto del equipo está organizado en cuatro turnos compuestos por: 10 técnicos de enfermería, cuatro asistentes de servicio y ocho matronas(es) (en Chile existe un programa de entrada directa -no a través de enfermería- de 5 años de educación universitaria). Todos los equipos rotan en turnos de día y de noche, y luego tienen dos días libres (después de lo cual se repite el ciclo). El personal de seguridad, limpieza, alimentación y mantenimiento de la unidad es parte de una sociedad concesionaria.

A pesar de haber abierto a fines de 2014, la maternidad comenzó a trabajar en pleno en 2016, cuando las salas integrales de parto se convirtieron en funcionales y se implementó el modelo de “Parto Personalizado Seguro". Este modelo busca mejorar la satisfacción de las mujeres, reducir las intervenciones excesivas e innecesarias (incluidas las cesáreas), y mejorar los resultados maternos, fetales y neonatales. Desde 2018, la administración de la unidad ha incluido un análisis detallado y mensual de los nacimientos, de acuerdo con el lugar de desarrollo del trabajo de parto. Esto se hace para promover los mismos estándares de cuidado de las salas integrales de parto en las salas tradicionales, como la presencia de un acompañante durante el parto, la libre circulación y el caminar, el uso de métodos de alivio de dolor no farmacológicos, contacto de piel a piel de 30 minutos o más, lactancia materna a libre demanda del recién nacido, y la reducción de intervenciones como el uso de oxitocina sintética y la realización de episiotomía. Entre mayo de 2016 y de julio de 2020, ocurrieron 3,681 nacimientos en las salas de atención integrales de parto. Durante 2020 (hasta finales de julio), el 30% de todos los nacimientos del hospital ocurrieron en estas salas.

Atención y cuidados maternos durante la pandemia

Anticipando la llegada de la pandemia por COVID-19 a Chile, a principios de febrero, todo el personal del Hospital Dra. Eloísa Díaz comenzó a recibir capacitación en cursos de 27 horas sobre las precauciones estándar para la prevención de infecciones y el uso de EPP, como delantales desechables, guantes, mascarillas y protectores faciales. Los cursos se centraron en proteger al personal de salud y a las personas usuarias de la infección, y no en temas técnicos relacionados con el embarazo y el parto.

El 10 de marzo, se publicaron las últimas fechas disponibles para esta capacitación, cuando el país estaba a una semana de ingresar a la Fase 4 (de propagación sostenida del virus), y La Florida de ser declarada en estado de emergencia. Para esa fecha había poca información sobre el impacto de COVID-19 en mujeres embarazadas y en trabajo de parto, y algunas de las primeras orientaciones que habían sido publicadas en el mundo, recomendaban el retiro de algunos estándares de atención que se habían convertido en derechos en la maternidad (acompañamiento a elección durante el parto, contacto de la piel a la piel después del nacimiento, lactancia materna de inicio temprano) (Favre et al., 2020).

El personal de salud de la maternidad informó que el principal sentimiento durante esas primeras semanas era de miedo: a lo desconocido, de infectarse, de infectar a sus familiares. Las jefaturas de turno expresan: “Lo primero que se me viene a la cabeza es miedo. En el fondo nadie sabía mucho a que se iba a enfrentar al principio había mucho miedo de contagiarse” (1); “Generó miedo al principio, porque no sabíamos a qué nos estábamos enfrentando. Miedo de infectarnos, de infectar a nuestras familias, a nuestras usuarias, de que se pudiera contagiar un recién nacido” (4); “Se sentía que todos estábamos asustados y eso fue bastante fuerte” (5); “Había mucho temor a lo desconocido, a cómo nos íbamos a ir enfrentando a este proceso” (6).

El lunes 16 de marzo, el día en que los municipios de Santiago y La Florida fueron declarados en estado de emergencia, fue enviado el primer correo electrónico desde la jefatura de la unidad a todo el equipo, reconociendo que el escenario no tenía precedentes a nivel nacional y mundial, y enumerando las primeras medidas a implementar: suspensión del cuarto turno regular, moviéndose a un esquema de 24 × 3 (un turno de 24 horas seguido de 3 días de descanso); evitar besos y saludos de mano, prohibición de llegar al hospital vistiendo el uniforme (obligación de vestirse con el uniforme dentro del hospital); y suspensión de las vacaciones y cursos de capacitación. Una de las matronas entrevistadas (jefa de turno) señala:

> Respecto a la gestión, siento que mis jefes entendieron cómo se es jefe, cómo se es líder, con enfoque en las personas que trabajaban con ellos; supieron canalizar muy bien el miedo que teníamos y hacer todo posible por canalizar el miedo. Fueron finalmente un poco matrones, siento yo; como que tomaron al personal, lo educaron, tomaron todas las precauciones para que nosotros nos sintiéramos seguros trabajando. (1)

El 17 de marzo, en una reunión entre el CR de la mujer y el personal de la Unidad de Neonatología, comenzaron a diseñarse las "Medidas para prevenir la infección por COVID-19 en mujeres embarazadas y sus recién nacidos", en base a las primeras recomendaciones de la Sociedad Chilena de Pediatría. En la reunión, se afirmó claramente que esas primeras medidas, que eran muy conservadoras (no recomendando el contacto piel a la piel o la lactancia materna para las mujeres COVID-19$^+$), se modificarían a medida que se dispusiera de más evidencia. Dos días después, el Servicio de Salud Metropolitano Sur Oriente (SSMSO), que gestiona y coordina tres grandes hospitales, y la atención primaria de salud correspondientes al mismo territorio, emitió recomendaciones para la organización del sistema y el uso de EPP. Se creó el grupo de WhatsApp "COVID-19 SSMSO". La coordinadora de matronería destaca la creación de

esta red como uno de los hitos del proceso de manejo y respuesta:

> La comunicación, pero no solo entre los equipos internos sino intersectorial, entre el equipo interno y los equipos del extrasistema -APS, Servicio de Salud y Nivel Central-; eso es fundamental para poder consensuar ideas y a la vez que podamos ayudarnos. (6)

Solo tres días después, el 22 de marzo, tuvimos en la maternidad a la primera mujer embarazada con sospecha de COVID-19. Este caso gatilló las alarmas respecto de la posibilidad de cuarentenas preventivas de una gran parte del personal, y nos hizo organizar al personal de una manera que no impactara los estándares de atención, y que al mismo tiempo, no implicara una sobrecarga de trabajo demasiado grande a los equipos. En ese día, y coincidiendo con el inicio de un toque de queda nacional, se decidió que se suspenderían todas las visitas a las usuarias hospitalizados, incluido el acompañamiento durante el trabajo de parto y el parto.

El 3 de abril, dos miembros de nuestro personal comunicaron la presencia de síntomas COVID-19 y se tomaron la prueba en la unidad de emergencia del hospital; esta fue la primera vez que nos vimos obligados a revisar el flujo del personal. Afortunadamente, ambas pruebas salieron negativas. Esta experiencia condujo a la creación de un grupo ejecutivo de WhatsApp compuesto por los cuatro jefes de turno, los dos supervisores y el coordinador de maternidad -todas(os) matronas(es)- como alternativa a un grupo existente y más extenso que incluía a otros niveles de supervisión.

Al día siguiente, decidimos que el uso de mascarillas era obligatorio durante todos los turnos y durante todas las interacciones entre el personal y las personas usuarias. Estando muy en desacuerdo con la suspensión del acompañamiento durante el parto, comenzamos a avanzar en acciones para revertir esta medida. Desde allí, solicitamos la provisión de mascarillas quirúrgicas para todo el personal, y también para todas las mujeres y sus acompañantes. Con respecto a esta forma de gestión participativa, una jefa de turno expresa:

> Otra cosa que me llama mucho la atención es cómo funcionamos como equipo, siento que cada decisión que se ha tomado siempre ha incluido la opinión de los jefes de turno, y eso me parece bueno en lo productivo y también porque es un tema de respeto y valoración con tu trabajo (…) Cada medida que se adopta está hecha por un supervisor pero vista por un equipo clínico; lo hace más fácil porque están las colaboraciones ahí. (3)

En el recién creado grupo de WhatsApp, comenzamos a discutir inmediatamente la mejor manera de organizar al personal. El 13 de abril se presentaron tres propuestas y los responsables de la unidad se inclinaron por el rotativo 24x4, que implicaba dividir los cuatro turnos existentes y crear un quinto. Para reducir el riesgo de infección, habría menos personal en cada turno (dos técnicos de enfermería, un asistente de servicio y una matrona menos). Se contrató una nueva matrona para completar estos turnos, este nuevo modelo organizacional fue oficializado cuatro días después, y comenzó a operar el lunes 20 de abril. El correo electrónico que comunicó estas medidas explicaba: "hemos estado trabajando en estas propuestas, junto con las cuatro jefas de turno, durante más de 10 días para poder afrontar de la mejor forma posible los escenarios a los que nos podamos enfrentar". Con respecto a estas decisiones, una jefa de turno afirma que:

> Las gestiones han sido muy oportunas; siento que al principio costó un poco agarrar vuelo y decidirse a hacer cambios, hubo un punto en el que hubo que saltar y hacer cambios grandes, como agregar un turno, y se hicieron los cambios que había que hacer. Creo que desde ahí en adelante las gestiones han sido super oportunas y asertivas, y nos han hecho las cosas mas fáciles, y para el equipo, mas tranquilizadoras. (2)

En el mismo correo se insistió en el uso de mascarillas N-95 en todas las áreas de la unidad, y se solicitó a las jefaturas de turno y a todos los miembros de la unidad, que supervisaran dicha indicación. En cuanto a los canales de comunicación e información utilizados, las jefaturas de turno señalan lo siguiente:

> Que se entregue la información es fundamental porque eso ayuda a bajar la ansiedad que se produce en un contexto así (…) La comunicación directa de las jefaturas de turno, y las jefaturas de la unidad es algo que también destacan como un elemento facilitador. (5)

> Lo que lo hace más fácil de llevar es la conversación tan directa que tenemos entre las cinco jefes de turno y los supervisores, yo sé que tengo en línea directa para conversar y buscar una solución. (3)

> Me deja tranquila que los canales de comunicación siempre han estado abiertos. A pesar de que quizás yo no tenga aquí y ahora una información particular, tengo la tranquilidad de que si necesito esa información puedo preguntarla y si alguien de mi equipo no la sabe, mis jefes sabrán; y si los jefes no la saben yo se que van a hacer todo lo posible por tenerme la respuesta. (1)

En cuanto a las infecciones de los miembros del personal, entre abril y julio tuvimos ocho casos de COVID-19, de los cuales al menos tres se infectaron fuera del hospital (Tabla 1); estos números son bajos en comparación con las otras unidades dentro del hospital. El ginecobstetra jefe explica cómo la unidad se anticipó con estas medidas:

> Yo creo que nosotros nos adelantamos a las medidas ministeriales. Principalmente el equipo de matronas, la coordinadora y el supervisor, se tomaron muy seriamente la situación, y se empezaron a tomar las medidas de prevención mucho antes. Nosotros empezamos a usar mascarilla en forma obligatoria antes de que el ministerio lo dijera. (8)

TABLA 1. Personal de salud de la unidad de maternidad en cuarentena preventiva y con test (+) para COVID-19 entre abril y julio de 2020 (N° total de personas que trabajan en la unidad=103).

	COVID-19	Cuarentena preventiva
Abril	1	19
Mayo	3	2
Junio	2	4
Julio	2	1
Total	8	26

Basado en la información del Centro de Atención Integral del Funcionario (CAIF), Hospital Dra.Eloísa Díaz.

El camino desde la suspensión al restablecimiento de los estándares de cuidado

La medida de la suspensión del acompañamiento materno del 22 de marzo golpeó fuerte a los equipos. Así dieron cuenta algunas jefas de turno:

> Siento que no hay justificación válida para tener que decirle eso a una mujer; tener que decírselo, es como predicar una cuestión con que no estas de acuerdo, no sé, no se bien como plasmarlo en palabras, solo que fue muy terrible. (1)

> Era terrible. Porque nosotros estábamos acostumbrados a que nuestras mujeres estuvieran en su trabajo de parto, en el parto y en la recuperación siempre acompañadas. Y en un principio cuando no tenían este acompañamiento era como angustiante, generaba una sensación muy triste que no estuvieran acompañadas. (5)

Es interesante notar el reconocimiento del equipo de que el miedo jugó un papel central en la toma de decisiones durante el comienzo de la pandemia. Una de las jefas de turno lo relata de la siguiente forma:

> Abusamos un poco del miedo, del hecho del desconocimiento, lo que nos llevó a tomar medidas exageradas que quizás no eran tan necesarias y que se podrían haber evitado. (3)

Los sentimientos de desaprobación de las medidas se expresaron en el grupo de WhatsApp de las jefaturas de turno durante los últimos días de marzo, lo que llevó a discutir la factibilidad de desarrollar un protocolo para volver a admitir acompañantes. Las opiniones fueron unánimes, y el 31 de marzo se presentó el primer borrador del protocolo, que finalmente se puso en funcionamiento el 9 de abril. La coordinadora de la unidad relata:

> Se buscó evidencia, se trabajó en conjunto con la unidad neonatal, se vio cuáles eran las mejores recomendaciones. (…) El hospital nos dio el apoyo y nos entregó todos los insumos que necesitábamos para poder cumplir con el protocolo. Se sacó en tiempo record, se analizó por matronería, equipo médico, jefatura, neonatología. Pasó por los distintos entes para que todos pudieran opinar y decir qué era mejor y qué incorporar. (…) Fue bastante positivo, y se dio más que nada por el impulso, las ganas del equipo de siempre estar haciendo cosas que permitan favorecer el respeto permanente por los derechos de la mujer. (6)

Una jefa de turno agrega:

> El equipo abogó por los derechos de las familias, y por otra parte, los supervisores y jefaturas de más arriba estuvieron dispuestos a escuchar y ver que estaban los recursos que se necesitaban para permitir que esto se pudiera mantener. (3)

Este protocolo también abordó el contacto piel a piel en casos de mujeres con sospecha o confirmación de COVID-19. Se utilizó como referencia la tercera versión del protocolo de la Sociedad Chilena de Pediatría (del 2 de abril); que recomendaba el contacto piel con piel y la lactancia materna en casos sospechosos o confirmados, a menos que el recién nacido fuera prematuro o la enfermedad de la mujer no lo permitiera. Se restableció el acompañamiento durante el parto, el contacto piel con piel y la lactancia. Sin embargo, las(os) matronas(es) informan sobre otros impactos negativos que ha tenido la pandemia en la calidad de la atención brindada, como los efectos del uso de EPP, como señala una matrona:

> Además hay una cuestión media física, de que estamos atendiendo a las mujeres tapados enteros. Estamos poniendo una barrera física de guantes, mascarilla, gorro, plástico, y es súper heavy no poder tocar a una mujer en trabajo de parto que requiere ser tocada, que tú sabes que las puedes apoyar moviéndola, y hacer todo esto a través del plástico. Esto puede sonar simbólico o hasta medio poético, pero no es lo mismo. (1)

Cuando la comunidad no puede ir al hospital, el hospital va a la comunidad a través de teléfonos celulares

La forma más habitual de contacto de la maternidad con la comunidad es a través de una visita guiada por sus instalaciones, que realizan las embarazadas y sus parejas hacia la semana 36 de gestación. Antes de la pandemia, este recorrido se realizaba todos los lunes, miércoles y viernes a las 11 de la mañana: una matrona mostraba las instalaciones y explicaba el proceso de admisión y atención durante el parto. Producto de la pandemia, estos recorridos fueron suspendidos y el equipo de salud tuvo la idea de realizar una "visita guiada virtual" a través de la cuenta de Instagram del hospital. El 22 de abril se realizó la primera visita guiada por Instagram, con gran éxito: más de 100 personas conectadas. La conciencia de la incertidumbre que generaba el escenario pandémico para las mujeres embarazadas llevó al equipo a solicitar autorización para crear una cuenta de Instagram, independiente de la del hospital, para la maternidad. La médica jefa de residentes señala: "La creatividad del equipo de matronas ha sido increíble, nos ha distinguido como maternidad" (7).

El 29 de abril se confirmó desde la dirección del hospital la autorización para crear nuestra cuenta de Instagram, reconociendo que la iniciativa había tenida buena acogida tanto en la comunidad externa como en los equipos internos. Una jefa de turno señala:

> Un gran hito es la creación del Instagram donde nos hemos podido contactar con la comunidad desde otro lugar, hemos hecho hartas actividades: lives, posts,

> responder preguntas y comentarios, hacer de nexo entre el hospital y APS, o entre el hospital y pacientes que se han ido de alta, etc. Además también hemos conocido el feedback directo de la gente, que es esencial para mejorar nuestro actuar. (2)

El 15 de mayo se realizó la primera publicación de Instagram y se comenzó a subir contenido relacionado con la “Semana Mundial del Parto Respetado”. Además de un primer ciclo de “lives” de Instagram en torno a esa celebración, se han realizado otros tres ciclos (Diálogos perinatales, Semana mundial de lactancia materna y Diálogos de matronería). Cuando estábamos completando el primer borrador de este artículo, el 31 de agosto de 2020, la cuenta @maternidaddraeloisadiaz tenía más de 3,240 seguidores, de los cuales el 92% eran mujeres y 8% hombres. El 56% de los seguidores de la cuenta tenían entre 24 y 35 años, seguido del grupo de 18 a 24 años. Una de las matronas a cargo del proyecto declara:

> Me motivó el poder crear nexos y redes tanto con los usuarios como con otras instituciones de salud nacionales e internacionales, y poder conectarnos mejor dentro de nuestra propia red de Servicio Sur-Oriente con otros CESFAM y otros hospitales. (I.3)

TABLA 2. Mensajes directos recibidos en la cuenta Instagram @maternidaddraeloisadiaz entre el 4 de mayo y el 31 de julio, 2020.

Variable	Número	%
Número de usuarios	228	100
La Florida	150	65.7
Otro	43	18.8
Indeterminado	35	15.3
Número de interacciones	434	
La Florida	335	77.1
Otro	61	14
Indeterminado	29	6.68
NA	9	2.07
Tipo de interacción		
Consultas	374	86.1
Consultas RPO/TDP	53	
Otras	321	
Felicitaciones	46	10.5
Reclamos	5	1.1
NA	9	2.0
Promedio de interacciones por usuario (todos)	1.9	
Promedio de interacciones por usuario (La Florida)	2.23	
Promedio de interacciones diarias (todos)	4.9	
Promedio de interacciones diarias (La Florida)	3.8	
Respuestas		
Si	423	97.5
No	11	2.5
Resolución de interconsultas	5	1.1

Un equipo de seis matronas voluntarias responde las preguntas de la comunidad las 24 horas del día, los 7 días de la semana. En cuanto a sus motivaciones, expresan: “Siento que estar en contacto directo con las usuarias o con sus familiares nos hace estar más cerca de las personas” (I.1); “Nuestro objetivo es disminuir la angustia en las gestantes, evitar que salgan, evitar la exposición innecesaria a contagio” (I.2); “Como el sistema de salud está organizado de esta forma tan jerárquica, era difícil lograr esta cercanía con la población por los canales tradicionales” (I.3); “Me interesó como un canal de información, y de información actualizada, como forma de tratar de generar pequeños cambios” (I.4). El equipo de matronas voluntarias de la cuenta Instagram señala la importancia y el impacto positivo que esta iniciativa ha tenido:

> Creo que es súper importante para bajar la ansiedad y sentirse seguras del lugar donde van a llegar; que somos seres humanos cercanos, que no es como tener un rango, o hacer una distinción entre yo soy el profesional y tú eres el paciente. (I.1)

> Las mujeres expresan que se sienten mucho más tranquilas, saben también que tienen una ventana abierta, que se pueden comunicar con nosotras de una manera expedita, saben que contestamos de noche, incluso en horas muy tarde. (I.2)

> El impacto es muy positivo de cercanía con el hospital, ha calmado las ansias increíblemente respecto al COVID, los ha mantenido mucho más informados. Se ha generado casi como un cariño hacia la maternidad, aman al equipo, aman la forma que tenemos de trabajar. (I.4)

Desde la apertura de la cuenta Instagram el 4 de mayo y hasta el 31 de julio, se realizaron un total de 53 publicaciones y 17 lives. En el mismo periodo las matronas respondieron las dudas de 228 usuarios de Instagram, en 434 interacciones (diálogo de dos o más mensajes entre una usuaria y una matrona voluntaria). El 77% de las interacciones ocurrió con mujeres de la comuna de La Florida. Así, en promedio, durante los primeros 88 días de funcionamiento de nuestra cuenta de Instagram, hubo 4.9 interacciones diarias con los usuarios, de las cuales 3.8 fueron con mujeres de La Florida (Tabla 2). Del total de interacciones durante este período, el 86% correspondió a preguntas, y el 11% a felicitaciones por la utilidad del Instagram de la maternidad y por su trabajo durante el brote de COVID, como: “Quiero felicitarlos por la página y por la información que comparten”, publicada el 29 de mayo, y “¡Gracias por sus lives en Instagram! Me siento empoderada e informada para mi parto con mis 39 semanas de embarazo ... ¡abrazos virtuales!" publicado el 14 de agosto. Con respecto a las experiencias de parto en la maternidad, el 31 de mayo, una madre escribió:

> Gracias por todo. Gracias por acompañarme en este hermoso momento. A pesar de todo lo que está sucediendo, nos sentimos cuidados como si no pasara nada malo, recibimos la mejor atención imaginable. Nunca dejaré de agradecer todo el apoyo y la preocupación por mí y por mi hija.

El 30 de julio, otra mujer que había dado a luz recientemente en la maternidad comentó:

> Muchas gracias por el tremendo trabajo que hacen en la maternidad. Gracias a la matrona, a sus palabras y su cariño, nació nuestra hija. Como pareja, vimos la mayoría de los lives de Instagram que organizó la maternidad y realmente nos ayudaron mucho a prepararnos para el parto. Gracias por la dedicación, pasión y amor que dan en cada nacimiento y nueva vida.

Los estándares de cuidado de la maternidad durante el brote COVID-19

Hemos descrito nuestro proceso de toma de decisiones como equipo de la maternidad desde el inicio de la pandemia COVID-19 en Chile, hasta el 31 de julio de 2020. Las primeras mujeres COVID-19 ingresaron a nuestra maternidad en mayo de 2020, luego de haber implementado los protocolos para proteger los estándares de cuidado pre pandémicos. A pesar de que el acompañamiento durante el parto fue suspendido durante los últimos 10 días de marzo y los primeros 10 días de abril (período en el que no tuvimos mujeres COVID-19^{+} en trabajo de parto), esta decisión tuvo un gran impacto en nuestros indicadores: nuestro 90% de acompañamiento durante el trabajo de parto y parto (2019) bajó a 81.4% entre enero y julio de 2020, y a 70% entre marzo y abril.

Otro dato que destaca es la diferencia entre los partos por cesárea, que fueron el 27.8% del total de partos en 2019, y el 32.5% durante los primeros 7 meses de 2020 (Tabla 3). El ginecobstetra jefe de la maternidad explica este aumento de cesáreas:

> Tendremos que mirar hacia atrás en nuestras prácticas. Por ejemplo, las cesáreas, cómo ha aumentado, no solo en nuestra institución sino en todos los hospitales de la Región Metropolitana, porque nos relajamos. Dijimos: “La gente está estresada, no nos vamos a poner tan quisquillosos de por qué estamos realizando cirugías, no vamos a hacer vigilancia, no vamos a auditar”, y eso ha tenido el impacto de un aumento en nuestra tasa de cesárea en 7 puntos. Ha afectado mucho. (8)

Entre mayo y julio de 2020 se produjeron 620 nacimientos en nuestra maternidad. Cincuenta y cinco (8.9%) de estas mujeres dieron positivo para SARS-CoV-2 mediante RT-PCR (52/55 dieron a luz dentro de los 14 días posteriores al diagnóstico de COVID-19). Las características demográficas, clínicas y el resultado del parto de los casos de COVID-19 se muestran en la Tabla 4. Veintiún casos eran mujeres migrantes (39%) y 45 (82%) eran residentes de La Florida. Dos mujeres COVID-19^{+} tenían comorbilidades respiratorias (asma). Se diagnosticaron 54 infecciones en el tercer trimestre, 41 (75%) fueron asintomáticas; dos ingresaron a cuidados intermedios (terapia respiratoria no invasiva) y dos ingresaron en la UCI.

Todas las mujeres con COVID-19 grave (cuatro casos) tuvieron un parto prematuro por cesárea y la indicación de esta estuvo relacionada con la infección por SARS-CoV-2. Treinta y seis mujeres (65.4%) con síntomas no graves tuvieron un parto vaginal y 15 tuvieron una cesárea por indicaciones no relacionadas con COVID-19. Si bien estas 36 cesáreas fueron señaladas como “no relacionadas” con COVID-19, podemos decir que el contexto pandémico incidió en los criterios para realizar estas cirugías, ya que, durante la pandemia, el personal se volvió menos riguroso en la aplicación de los protocolos de cesárea de la maternidad, y en caso de duda sobre el progreso fisiológico del trabajo de parto, prefirieron practicar una cesárea de emergencia, ya que brindaba una mejor oportunidad para el uso adecuado del EPP.

Del total de mujeres con COVID-19, sólo 29 (52.7%) estuvieron bajo condiciones de aislamiento, y por tanto en una de las Salas de Atención Integral del Parto, con todas las medidas de seguridad establecidos para estos casos. Las 26 mujeres restantes fueron atendidas “a ciegas”, es decir en desconocimiento del resultado del examen de RT-PCR, que comenzó a ser tomado el día 15 de mayo, de manera rutinaria a todas las gestantes que se hospitalizaban. La demora en el procesamiento del examen era entre 48 y 72 horas, motivo por el cual en muchos casos, recién se supo del diagnóstico en el posparto, y por tanto no fueron tratadas con las medidas más estrictas de aislamiento en una sala habilitada para aquello. La única opción de ingresar a unidad de aislamiento era tener PCR(+) conocido, presentar sintomatología, o haber sido referida como contacto estrecho.

En este grupo, los estándares que se ofrecieron se pueden ver en la Tabla 5. Quince mujeres estuvieron acompañadas, y se destaca que del total de 14 partos vaginales, de los cuales 12 (86%) fueron asistidos por matronas(es), el contacto piel a piel después del nacimiento durante al menos 30 minutos (que es el tiempo mínimo recomendado en los protocolos nacionales) alcanzó el 86%, por encima del promedio en 2020 (70%). A menudo, este contacto piel con piel duraba mucho más. Esto último se explica por los esfuerzos realizados por los equipos de salud para promover los derechos de nacimiento incluso en estos casos.

TABLA 3. Principales resultados obstétricos de todos los partos en la Maternidad durante 2019 y 2020 (hasta el 31 de julio).

	2020 (enero – julio)		2019 (enero – diciembre)	
	N	%	N	%
Total	1,518	100	3,003	100
Partos vaginales	939	60.9	1975	65.7
Fórceps	81	5.33	192	6.39
Cesáreas	494	32.5	836	27.8
Contacto piel a piel >30 min.	1,064	70.9	2110	70%
Lactancia precoz	571	37.6	1,197	39.8
Acompañamiento durante preparto y parto	1,236	81.4	2717	90.4
Apgar <7 a los 5 min	14	0.9	22	0.7

Basado en informes mensuales de la Unidad de Ginecología y Obstetricia.

TABLA 4. Características demográficas, clínicas y resultados perinatales de mujeres COVID-19+ por RT-PCR (n 55).

	(n) /[a] promedio	% [intervalo]
Nacionalidad		
Chile	34	61%
Venezuela	8	14%
Haití	6	11%
Colombia	2	4%
Perú	2	4%
Otra: Argentina, Bolivia y República Dominicana	3	6%
Residencia (comuna)		
La Florida	45	82%
Puente Alto	3	5%
La Granja	2	4%
La Pintana	2	4%
Otra (3)	3	5%
Edad materna (años)	29.4/[a]	[15–41]
Paridad		
0	21	38%
1	19	35%
2	10	18%
3 o más	5	9%
Trimestre de embarazo al diagnóstico	0	—
Primero	1	25+0
Segundo	54	[28+1–41+0]
Tercero	2	3.6%
Comorbilidades respiratorias	0	—
Síntomas		
No	41	75%
Fiebre	3	6%
Tos	4	7%
Disnea	5	9%
Otro	3	6%
Vía de parto		
Vaginal	36	65.4%
Cesárea	15	27.3%
Cesárea por condición materna COVID-19	4	7.3%
Admisión UCI	2	3.6%
Admisión cuidados intermedios	2	3.6%
Edad gestacional al parto (semanas)		
Pre término extremo (< 28 semanas)	1	1.8%
Pre término (< 34 semanas)	2	3.6%
Pre término tardío (34 – 36 semanas)	6	10.9%
Término temprano (37 – 38 semanas)	18	32.7%
Término (39 – 40 semanas)	25	45.5%
Término tardío (41 semanas)	3	5.5%
Post término (42 semanas y más)	0	—
Mujeres que tuvieron su parto dentro de 14 días después del diagnóstico de COVID-19 (RT-PCR)	52	94.5%

TABLA 5. Características clínicas, resultados perinatales y estándares de cuidado de mujeres COVID-19 que tuvieron su parto en condiciones de aislamiento (n 29).

	Vaginal n = 14 48%	Cesárea n = 11 38%	Cesárea (indicación por COVID-19) n = 4 14%
Edad materna (años; promedio, rango)	31.1 [18–40]	29.4 [22–41]	33.3 [30–36]
Síntomas (número; porcentaje)	2 (14%)	1 (9%)	4 (100%)
Edad gestacional al parto			
Pre término (<34 semanas)	0 (0%)	0 (0%)	2 (50%)
Pre término tardío (34–36 semanas)	1 (7%)	0 (0%)	2 (50%)
Termino (>37 semanas)	13 (93%)	11 (100%)	0 (0%)
Admisión UCI	0	0	2 (50%)
Peso nacimiento (gramos; promedio, rango)	3,144 [2,710 – 3,720]	3,032 [2,180 – 3,480]	2,291 [1,760 – 3,245]
Apgar 5 min > 7	14 (100%)	11 (100%)	2 (50%)
Mujeres acompañadas durante el parto (por pareja o persona significativa)	11 (79%)	4 (36%)	0 (0%)
Contacto piel a piel (30 min o más)	12 (86%)	3 (27%)	0 (0%)
Parto asistido por matrona	12 (86%)	0 (0%)	0 (0%)

DISCUSIÓN

En este artículo buscamos describir la respuesta a la pandemia COVID-19 en la maternidad pública Eloísa Díaz. Como hemos expuesto, esta maternidad ya se destacaba por altos estándares de cuidado desde antes de esta crisis sanitaria, implementando un modelo humanista de atención del parto como lo describe Davis-Floyd (2001), Davis-Floyd (2018), y fue capaz de mantener casi los mismos estándares durante los primeros meses del brote por COVID-19 (marzo a julio de 2020).

En la maternidad de La Florida, durante el 2019, el 70% de las usuarias tuvo contacto piel a piel inmediato de 30 minutos o más después del nacimiento, incluyendo lactancia precoz, y durante el 2020 (hasta fines de julio) esta cifra fue de 71%. El noventa por ciento de los trabajos de parto y partos contaron con acompañamiento en 2019, lo que disminuyó a 81% en el mismo período de 2020 (Tabla 3). Pero, como hemos mostrado, esta caída refleja la prohibición del acompañamiento que se produjo por solo 20 días, durante los cuales las(os) matronas(es) de la maternidad presionaron por protocolos para revertir la medida. Una vez que se compartieron las propuestas para restablecer los estándares de atención previos, todas las jefaturas mostraron apoyo. Esto demuestra la fuerza de una cultura colectiva de respeto a los derechos de la mujer y el recién nacido arraigada en el personal de la maternidad, así como la del incumplimiento de medidas que no cuentan con evidencia de sustento. Como lo expresa una de las jefas de turno de matronería: “Había ciertos estándares de cuidado que no podíamos dejar pasar, como la suspensión del contacto piel con piel, del acompañante durante el parto; no íbamos a normalizarlo simplemente porque alguien te lo dijo o porque ‘pareciese’ ser lo mejor".

Uno de los temores de nuestro equipo de salud era que la tasa de partos por cesárea aumentaría durante la pandemia, como se había informado en varios países y contextos en abril. Una revisión sistemática que incluyó 108 partos de mujeres diagnosticadas con COVID-19 mostro que el 91% tuvo su parto por cesárea (Zaigham and Andersson, 2020). Un estudio en España con 82 mujeres COVID-19^{+} mostró una tasa de cesárea del 47% (Martínez-Perez et al., 2020). En junio, los hallazgos de una encuesta global rápida en línea de profesionales de la salud materna y neonatal que hacían frente a la pandemia de COVID-19, y que incluyó respuestas de 81 países, mostraron que las cesáreas se realizaban frecuentemente en mujeres diagnosticadas con COVID-19, y que en algunas instalaciones tenían como objetivo reducir la duración del trabajo de parto y el tiempo dentro de las salas de parto, realizando cesáreas incluso en mujeres no infectadas (Semaan et al., 2020). En nuestra maternidad, en cambio, la tasa de partos por cesárea fue del 34.6% en el grupo de 55 mujeres con COVID-19, un 6.8% superior a la de 2019 (Tablas 3 y 4). Aunque se trata de una tasa superior a nuestros estándares pre pandémicos, sigue siendo más baja que la mayoría de las maternidades públicas en el país, que tuvieron un promedio de cesáreas del 41% en 2015 (National Institute of Human Rights, 2016). El personal de la maternidad ha reconocido este aumento y está trabajando en formas de revertirlo.

Las restricciones a las visitas presenciales a la maternidad y la dificultad para acceder a información actualizada sobre los protocolos de la misma, contribuyeron a incrementar la ansiedad que ya vivían las gestantes y sus familias debido a la pandemia, lo que motivó a buscar nuevas formas de contacto con ellas. Una editorial publicada en Women & Birth (Matvienko-Sikar et al., 2020) reconoce que la pandemia de COVID-19 ha creado el contexto para un aumento de experiencias de angustia relacionadas con: restricciones en las rutinas normales; preocupaciones sobre el riesgo de infección; cambios en la atención prenatal y en el acceso a la atención de salud perinatal; restricciones a la presencia de parejas durante el cuidado de la maternidad; así como un acceso reducido a las redes de apoyo. En este escenario, el apoyo de las redes y de matronas(es), así como de otros profesionales de la salud, es crítico para la salud mental de las mujeres durante la pandemia. En nuestra maternidad, la conciencia de esta mayor vulnerabilidad de la salud mental de nuestra comunidad nos llevó a crear la cuenta de Instagram como canal de comunicación y a realizar “visitas virtuales” por nuestras instalaciones. Este canal fue administrado hasta finales de julio por seis matronas voluntarias; durante agosto, aumentaron a 10. Estas voluntarias continúan interactuando con la comunidad de manera continua, y desde ya anticipamos que este canal de comunicación debe fortalecerse y tiene un gran potencial en el futuro post pandémico.

Otro elemento relevante que debemos mencionar es la creación, el 23 de julio, de la “Mesa de Género” del Hospital Dra. Eloísa Díaz, que ha iniciado una campaña de concientización

para rechazar cualquier práctica o conducta que viole los derechos de la mujer dentro del hospital (Dra. Eloísa Díaz Hospital, 2020). Entre los miembros de esta iniciativa sin precedentes, se encuentran una matrona (una de nuestras voluntarias de Instagram) y una médica ginecobstetra de nuestra maternidad.

La gran conciencia de los derechos de las mujeres y de las comunidades a una atención médica digna ha sido facilitada por nuestro personal de matronas(es), que son predominantemente jóvenes (en sus 30´s) y que han participado activamente en el movimiento social por una mayor igualdad y dignidad que ha movilizado a Chile durante los últimos 15 años. Muchas de las personas integrantes de nuestro equipo formaron parte del movimiento estudiantil denominado la "Revolución Pingüina" de 2006, que exigía una educación de calidad y gratuita, y de la masiva protesta social de octubre de 2019 contra la desigualdad y las necesidades no resueltas de la población por mejorar la educación, pensiones y salud, como expresa claramente una matrona:

> El hecho de que nuestra maternidad tenga equipos jóvenes ahora mismo, nos resultó muy, muy beneficioso. Somos una generación que siempre ha estado sujeta a cambios, lo que ha actuado como factor protector para nosotros: el hecho de que podemos adaptarnos, de que estamos acostumbrados al cambio, que somos una generación de cambios, que hemos exigido cambios en nuestro país. Estamos acostumbrados, fuimos la generación de los "pingüinos", fuimos parte de las huelgas estudiantiles, hemos sido parte de la revuelta de octubre. Esto significa que creemos en nuestra capacidad para cambiar las cosas, no ahogarnos en los problemas sino enfrentarlos y buscar, participar, involucrarnos, tratar de encontrar soluciones creativas... Esa es nuestra mayor fortaleza y lo que nos diferencia de otras instituciones. (3)

Además, el sentido de un propósito colectivo ha hecho que el equipo se vuelva más cercano y fuerte. En palabras de una jefa de turno: "Esto nos ha hecho ser más familia de lo que ya éramos" (3). La jefa de residentes de obstetricia y ginecología reconoce:

> Estas situaciones de emergencia afiatan a los equipos de trabajo. A pesar de todas las cosas que puedan pasar en el día a día, lo que hemos vivido ha sido lo mismo para todos, y todos hemos tratado de trabajar por una misma causa. Creo que eso es lo más importante. Yo siempre he dicho que nosotros somos un equipo, que no podemos trabajar sin matronas y técnicos, y eso se demostró en esta pandemia, que somos un equipo. (7)

Con este equipo joven y comprometido, con facilidad para adaptarse al cambio, con un profundo enfoque de género y derechos humanos que ha sido capaz de proteger los derechos de las mujeres y los recién nacidos durante la pandemia de COVID-19, esperamos liderar el camino para que otras maternidades en el país y en la región puedan seguir caminos similares, y esperamos unirnos a la *Iniciativa Internacional del Parto (ICI por sus siglas en inglés): 12 Pasos para una Atención de Maternidad Segura y Respetuosa*, en un futuro próximo (Lalonde et al., 2019; www. internationalchildbirth.com).

Finalmente, un tema de gran preocupación en la actualidad es la salud mental del personal de la maternidad, que se ha destacado al máximo en el contexto del COVID-19, como expresan nuestras jefaturas de turno: "El equipo está sometido a un estrés basal más alto, y eso ha tenido repercusiones sobre todo en la salud mental, las que se incrementarán en un futuro próximo" (2); "Un drenaje emocional, creo que es de lejos lo que más va a pesar. El estrés de estar en una situación en la que quizás no quieres estar, me refiero al estrés de que todo se basa en el miedo." (3). Esta preocupación por la salud mental es consistente con los resultados preliminares del estudio "El impacto de la pandemia COVID-19 en la salud mental de los trabajadores en los servicios de salud" en el país: de los 954 trabajadores de la salud entrevistados en todo el país, el 37.6% reporta falta de energía y fatiga, el 38.6% falta de apetito y, lo más preocupante, que el 31.4% presenta síntomas depresivos de moderados a altos. (Medical College of Chile, 2020).

DECLARACIÓN SOBRE DATOS

Los datos en bruto que respaldan las conclusiones de este artículo serán puestos a disposición por los(as) autores(as), sin reservas.

DECLARACIÓN ETICA

Los estudios con participantes humanos fueron revisados y aprobados por el Comité Ético Científico Hospital Dra. Eloísa Díaz, La Florida. Los pacientes/participantes proporcionaron su consentimiento informado por escrito para participar en este estudio.

CONTRIBUCIÓN DE LOS AUTORES

GL y MS: diseño del trabajo, adquisición de datos, análisis e interpretación de datos, redacción del trabajo, aprobación final. SQ y CL: adquisición de datos, análisis e interpretación de datos, redacción del trabajo. VF: adquisición de datos, diseño del trabajo, aprobación final. CS, SD y CF: diseño del trabajo, aprobación final.

AGRADECIMIENTOS

Reconociendo que la salud mental de nuestro personal es una de nuestras prioridades, estamos seguros de que hemos hecho y estamos haciendo todo lo que está a nuestro alcance para enfrentar la pandemia de COVID-19 de acuerdo con nuestros principios de atención. Nuestro compromiso para lo que suceda a continuación, una segunda ola o una futura pandemia, es continuar manteniendo los derechos de las mujeres y sus recién nacidos como nuestra *primera* prioridad.

REFERENCIAS

Binfa, L., Pantoja, L., Ortiz, J., Gurovich, M., Cavada, G., and Foster, J. (2016). Assessment of the implementation of the model of integrated and humanised midwifery health services in Chile. Midwifery 35, 53–61. doi:10.1016/j.midw.2016.01.018

Childbirth is Ours (El Parto es Nuestro) (2020). The childbirth is ours complaint about malpractice during COVID-19. Available at: https://www.elpartoesnuestro.es/blog/2020/04/05/el-parto-es-nuestro-denuncia-mala-praxispor-covid19 (Accessed August 9, 2020).

Chilean College of Midwives (2020). Midwives meeting health minister and raise urgent need for release a national guideline for women's health care in pandemic. Available at: https://colegiodematronas.cl/?p2901 (Accessed August 9, 2020).

Chilean Obstetric Violence Observatory (OVO Chile) (2018). Childbirth survey Chile. Available at: https://www.researchgate.net/publication/325933924_OVO_Chile_2018_Resultados_Primera_Encuesta_sobre_el_Nacimiento_en_Chile (Accessed August 8, 2020).

Chilean Obstetric Violence Observatory (OVO Chile) (2020). Public statement about COVID-19 and childbirth in Chile. Available at: http://ovochile.cl/declaracion-publica-sobre-covid-19-y-nacimientos-en-chile/ (Accessed August 9, 2020).

Chilean Pediatric Society (2020). Guidelines to prevent COVID-19 in newborn and its clinical management during pandemic. Available at: https://sochipe.cl/v3/ covid/27.pdf (Accessed August 8, 2020).

Creswell, J. (2014). Research design: qualitative, quantitative, and mixed methods approaches. Thousand Oaks, CA: SAGE Publications.

Davis-Floyd, R. (2001). The technocratic, humanistic, and holistic paradigms of childbirth. Int. J. Gynaecol. Obstet. 75 (Suppl. 1), S5–S23. doi:10.1016/S00207292(01)00510-0

Davis-Floyd, R. (2018). "The technocratic, humanistic, and holistic paradigms of birth and health care," in Ways of knowing about birth: mothers, midwives, medicine, and birth activism by Robbie Davis-Floyd. Long Grove, IL: Waveland Press, 3–44.

Dra. Eloísa Díaz Hospital (2020). Dra. Eloísa Díaz hospital gender equity round table. Available at: http://www.hospitallaflorida.cl/hospital-dra-eloisa-diaz-dela-florida-instaura-la-mesa-de-genero/ (Accessed September 9, 2020).

Economic Commission for Latin America and the Caribbean (ECLAC) (2012). Social protection systems in Latin America and the Caribbean: Chile. Available at: https://www.cepal.org/en/publications/4031-social-protection-systemslatin-america-and-caribbean-chile (Accessed August 8, 2020).

Favre, G., Pomar, L., Qi, X., Nielsen-Saines, K., Musso, D., and Baud, D. (2020). Guidelines for pregnant women with suspected SARS-CoV-2 infection. Lancet Infect. Dis. 20 (6), P652–P653. doi:10.1016/S1473-3099(20)30157

Goic, A. (2015). The chilean health care system: the task ahead. Rev. Med. Chile 143, 774–786. doi:10.4067/S0034-98872015000600011

Human Rights in Childbirth (2020). Human rights violations in pregnancy, birth and postpartum during the COVID-19 pandemic. Available at: http://humanrightsinchildbirth.org/wp-content/uploads/2020/05/Human-Rights-inChildbirth-Pregnancy-Birth-and-Postpartum-During-COVID19-Report-May2020.pdf (Accessed August 9, 2020).

International Confederation of Midwives (2020). Women's rights in childbirth must be upheld during the coronavirus pandemic. Available at: https://www.internationalmidwives.org/assets/files/news-files/2020/03/icm-statement_upholding-womens-rights-during-covid19-5e83ae2ebfe59.pdf (Accessed August 9, 2020).

John Hopkins University (2020). Mortality analyses, coronavirus resource center. Available at: https://coronavirus.jhu.edu/data/mortality (Accessed August 9, 2020).

Lalonde, A., Herschderfer, K., Pascali-Bonaro, D., Hanson, C., Fuchtner, C., and Visser, G. H. A. (2019). The international childbirth initiative: 12 steps to safe and respectful motherbaby-family maternity care. Int. J. Gynaecol. Obstet. 146 (1), 65–73. doi:10.1002/ijgo.12844

Martínez-Perez, O., Vouga, M., Cruz Melguizo, S., Forcen, L., Panchaud, A., Chapuli, M., et al. (2020). Association between mode of delivery among pregnant women with COVID-19 and maternal and neonatal outcomes in Spain. JAMA 324 (3), 296–299. doi:10.1001/jama.2020.10125

Martinez-Portilla, R., Goncé, A., Hawkins-Villarreal, A., and Figueras, F. (2020). A Spanish-translated clinical algorithm for management of suspected SARS-CoV2 infection in pregnant women. Lancet Infect. Dis. 20 (6), 655. doi:10.1016/ S1473-3099(20)30285-1

Matvienko-Sikar, K., Meedya, S., and Ravaldi, C. (2020). Perinatal mental health during the COVID-19 pandemic. Women Birth 33 (4), 309–310. doi:10.1016/j.wombi.2020.04.006

Medical College of Chile (2020). The COVID-19 health care workers study. Available at: http://www.colegiomedico.cl/primer-informe-the-covid-19health-care-workers-study-un-tercio-de-los-encuestados-presenta-sintomasdepresivos-de-caracter-moderado-a-grave/ (Accessed September 9, 2020). Ministry of Health (2020b). Epidemiological report N° 39. SARS-CoV2 infection – COVID-19 illness. Department of Epidemiology. . Available at: https://www. minsal.cl/wp-content/uploads/2020/08/Informe-Epidemiologico-39-MINSALAgosto.pdf (Accessed August 9, 2020).

Ministry of Health, (2020d). Guidelines for the management of SARS-CoV-2 (COVID-19) cases in pregnant and postpartum women and/or dyads. Available at: https://colegiodematronas.cl/wp-content/uploads/2020/06/Ord.-N%C2%BA-2388.pdf (Accessed August 9, 2020).

Ministry of Health. (2008). Manual for personalized attention in the reproductive process. Available at: http://web.minsal.cl/portal/url/item/795c63caff4fde9fe04001011f014bf2. pdf (Accessed August 8, 2020).

Ministry of Health. (2020c). Ordinance N° 1891: recommendations for postpartum women, boys and girls regarding risk factors of child morbidity and mortality, and their mental health in the COVID-19. Available at: http://www.crececontigo.gob.cl/wp-content/uploads/2020/06/ORD.-N%C2%B0-1891RECOMENDACIONES-PUERPERAS-NIN%CC%83OS-AS-RIESGO-DEMORBIMORTALIDAD-SALUD-MENTAL-EN-PANDEMIA-COVID-19. pdf (Accessed August 9, 2020).

Ministry of Health. (2020e). Vital Statistics, Chile. Registered live births by date and municipality. Available at: https://public.tableau.com/profile/deis4231#!/vizhome/HechosVitalesdesdeel2000/NotaTcnica?publishyes (Accessed August 8, 2020).

Ministry of Health. (2020a). Women's health program dashboard. Available at: http://www.deis.cl (Accessed August 8, 2020).

Ministry of Social Development and Family - World Bank (2018). 10 years of Chile grows with you (Chile Crece Contigo). Key components and lessons learned for the setting up of comprehensive child development support systems. Available at: http://www.crececontigo.gob.cl/wp-content/uploads/2020/08/A-10-añosChile-Crece-Contigo-claves-y-aprendizajes-para-la-construcción-de-sistemascomprehensivos-de-apoyo-al-desarrollo-infantil.pdf (Accessed August 9, 2020).

Ministry of Social Development and Family (MDSF) (2018). National household survey (CASEN) 2017. Available at: http://observatorio.ministeriodesarrollosocial.gob.cl/casen-multidimensional/casen/casen_2017.php (Accessed August 8, 2020).

Ministry of the Interior and Public Security (2020). Supreme decree no. 104. State of constitutional exception of catastrophe. Available at: https://www.bcn.cl/leychile/navegar?idNorma1143580 (Accessed August 9, 2020).

Municipality of La Florida (2020). Decree No1514. Available at: https://www.laflorida.cl/sitio/wp-content/uploads/2020/07/decreto-exento-1514.pdf (Accessed August 9, 2020).

Narang, K., Ibirogba, E., Elrefaei, A., Teles, A., Theiler, R., Nomura, R., et al. (2020). SARS-CoV-2 in pregnancy: a comprehensive summary of current guidelines. J. Clin. Med. 9 (5), 1521. doi:10.3390/jcm9051521

National Institute of Human Rights (INDH) (2016). Situación de los derechos humanos en Chile: informe anual 2016. Available at: http://www.indh.cl/informe-anualsituacion-de-los-derechos-humanos-en-chile-2016 (Accessed August 9, 2020).

National Institute of Statistics (INE) (2018). 2017 census data. Available at: https:// www.censo2017.cl/descargas/home/sintesis-de-resultados-censo2017.pdf (Accessed August 8, 2020).

National Institute of Statistics (INE) (2019). National vital statistics 2017. Available at: https://www.ine.cl/docs/default-source/nacimientos-matrimonios-y-defunciones/ publicaciones-y-anuarios/anuarios-de-estad%C3%ADsticas-vitales/anuario-de-estad %C3%ADsticas-vitales-2017.pdf?sfvrsn95e68aba_4 (Accessed August 8, 2020).

National Public Health Insurance Fund (FONASA) (2020). Income brackets 2020. Available at: https://www.fonasa.cl/sites/fonasa/conoce-fonasa (Accessed August 8, 2020).

Organization for Economic Cooperation and Development (OECD) (2020). OECD data. Health spending. Available at: https://data.oecd.org/healthres/health-spending.htm (Accessed August 8, 2020).

Richter, L., Daelmans, B., Lombardi, J., Heymann, J., Lopez Boo, F., Behrman, J., et al. (2016). Investing in the foundation of sustainable development: pathways to scale up for early childhood development. Lancet 389 (10064), 103–118. doi:10.1016/S0140-6736(16)31698-1

Sadler, M., Leiva, G., and Olza, I. (2020). COVID-19 as a risk factor for obstetric violence. Sex. Reprod. Health Matters 28, 1785379. doi:10.1080/26410397.2020. 1785379

Schmid, M., Fontijn, J., Ochsenbein-Kölble, N., Berger, C., and Bassler, D. (2020). COVID-19 in pregnant women. Lancet Infect. Dis. 20 (6), 653. doi:10.1016/ S1473-3099(20)30175-4

Semaan, A., Audet, C., Huysmans, E., Afolabi, B., Assarag, B., Banke-Thomas, A., et al. (2020). Voices from the frontline: findings from a thematic analysis of a rapid online global survey of maternal and newborn health professionals facing the COVID-19 pandemic. BMJ Global Health 5 (6), e002967. doi:10.1136/ bmjgh-2020-002967

Smith, H. (2020). Impact of COVID-19 on neonatal health: are we causing more harm than good? Eur J. Midwifery 4, 9. doi:10.18332/ejm/120245

South-East Metropolitan Health Service (2020). Good practices and innovation, second meeting. Available at: https://redsalud.ssmso.cl/destacados-proyectos-de-losestablecimientos-de-salud-del-sur-oriente-fueron-premiados-en-el-2-encuentro-debuenas-practicas-e-innovacion-del-ssmso/ (Accessed August 9, 2020).

Stuebe, A. (2020). Should infants be separated from mothers with COVID-19? First, do no harm. Breastfeed. Med. 15 (5), 351–352. doi:10.1089/bfm.2020. 29153.ams

United Nations Children's Fund (UNICEF) (2018). Levels and trends in child mortality. Report 2018. Available at: https://www.unicef.org/publications/ index_103264.html (Accessed August 8, 2020).

World Bank (2020). Data bank world development indicators. Available at: https:// databank.worldbank.org/source/world-development-indicators (Accessed August 8, 2020).

World Bank (2020). The World Bank in Chile. Available at: https://www. worldbank.org/en/country/chile/overview (Accessed August 8, 2020).

World Health Organization (WHO) (2020b). Clinical management of severe acute respiratory infection (SARI) when COVID-19 disease is suspected. Available at: https://apps.who.int/iris/handle/10665/331446 (Accessed August 9, 2020).

World Health Organization (WHO) (2019). Trends in maternal mortality 2000 to 2017: estimates by WHO, UNICEF, UNFPA, World Bank Group and the United Nations Population Division. Geneva, Switzerland:WHO. Licence: CC BY-NC-SA 3.0 IGO.

World Health Organization (WHO) (2020a). WHO COVID-19 homepage. Available at: https://covid19.who.int/region/amro/country/cl (Accessed August 9, 2020).

Zaigham, M., and Andersson, O. (2020). Maternal and perinatal outcomes with COVID-19: a systematic review of 108 pregnancies. Acta Obstet. Gynecol. Scand. 99 (7), 823–829. doi:10.1111/aogs.13867

Conflict of Interest: The authors declare that the research was conducted in the absence of any commercial or financial relationships that could be construed as a potential conflict of interest.

Copyright © 2021 Leiva, Sadler, López, Quezada, Flores, Sierra, Díaz and Figueroa. This is an open-access article distributed under the terms of the Creative Commons Attribution License (CC BY). The use, distribution or reproduction in other forums is permitted, provided the original author(s) and the copyright owner(s) are credited and that the original publication in this journal is cited, in accordance with accepted academic practice. No use, distribution or reproduction is permitted which does not comply with these terms.
